# Fungal Biodiversity in Salt Marsh Ecosystems

**DOI:** 10.3390/jof7080648

**Published:** 2021-08-09

**Authors:** Mark S. Calabon, E. B. Gareth Jones, Itthayakorn Promputtha, Kevin D. Hyde

**Affiliations:** 1 Center of Excellence in Fungal Research, Mae Fah Luang University, Chiang Rai 57100, Thailand; mscalabon@up.edu.ph; 2 School of Science, Mae Fah Luang University, Chiang Rai 57100, Thailand; 3 Department of Botany and Microbiology, College of Science, King Saud University, P.O. Box 2455, Riyadh 11451, Saudi Arabia; torperadgj@gmail.com; 4 Department of Biology, Faculty of Science, Chiang Mai University, Chiang Mai 50200, Thailand; itthayakorn.p@cmu.ac.th; 5 Environmental Science Research Center, Faculty of Science, Chiang Mai University, Chiang Mai 50200, Thailand; 6 Innovative Institute of Plant Health, Zhongkai University of Agriculture and Engineering, Guangzhou 510225, China

**Keywords:** halophytes, marine fungi, marine mycology, salt marsh fungi, worldwide distribution

## Abstract

This review brings together the research efforts on salt marsh fungi, including their geographical distribution and host association. A total of 486 taxa associated with different hosts in salt marsh ecosystems are listed in this review. The taxa belong to three phyla wherein Ascomycota dominates the taxa from salt marsh ecosystems accounting for 95.27% (463 taxa). The Basidiomycota and Mucoromycota constitute 19 taxa and four taxa, respectively. Dothideomycetes has the highest number of taxa, which comprises 47.12% (229 taxa), followed by Sordariomycetes with 167 taxa (34.36%). Pleosporales is the largest order with 178 taxa recorded. Twenty-seven genera under 11 families of halophytes were reviewed for its fungal associates. *Juncus roemerianus* has been extensively studied for its associates with 162 documented taxa followed by *Phragmites australis* (137 taxa) and *Spartina alterniflora* (79 taxa). The highest number of salt marsh fungi have been recorded from Atlantic Ocean countries wherein the USA had the highest number of species recorded (232 taxa) followed by the UK (101 taxa), the Netherlands (74 taxa), and Argentina (51 taxa). China had the highest number of salt marsh fungi in the Pacific Ocean with 165 taxa reported, while in the Indian Ocean, India reported the highest taxa (16 taxa). Many salt marsh areas remain unexplored, especially those habitats in the Indian and Pacific Oceans areas that are hotspots of biodiversity and novel fungal taxa based on the exploration of various habitats.

## 1. Introduction

Salt marsh ecosystems are known for their high productivity, exceeding primary production estimates of species rich ecosystems (e.g., tropical rainforests, coral reefs) [1]. The flora in salt marsh ecosystems is mainly composed of grasses, herbs, and shrubs and these are terrestrial organisms variously adapted to, or tolerant of, a semi-marine environment. Halophytes are a diverse group of plants that have a worldwide distribution, and grow in different climatic regions, wherein soils have high salinity levels [2]. Halophytes are common in temperate and Mediterranean climates, and fewer both in the tropics and at high latitudes [3,4,5,6]. The vegetation in these ecosystems shows the vertical zonation of different communities as tidal submergence decreases with increasing elevation, and species tolerance to changing gradient conditions. Salt marsh vegetation generally increases the attenuation of both tidal currents and waves as they pass over the vegetated area and immobilize elements with their sediments. Furthermore, halophytes serve as a natural buffer, protecting other shoreline ecosystems from human impacts and disturbances. The area provides a habitat and nursery for marine organisms [7]. Worldwide, salt marshes cover an area of 5,495,089 hectare in 43 countries [8].

**Figure 1:**
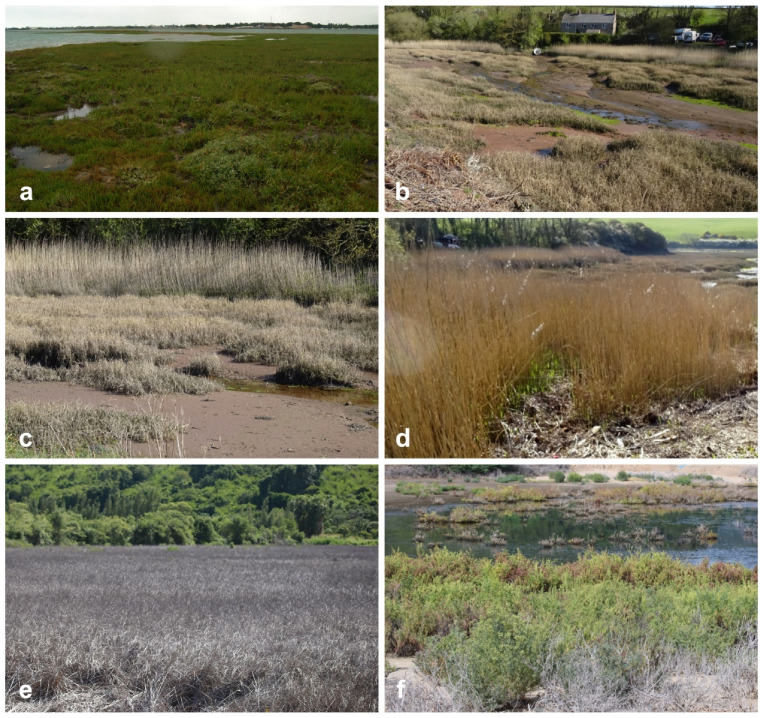
Salt marsh ecosystems in UK (**a**–**d**) and Thailand (**e**–**f**). (**b**–**d**) Tidal grasses, *Spartina townsendii* (Poaceae) and *Phragmites* (Poaceae), dominate the salt marsh in UK (50°49′55.4″ N 0°58′25.1″ W; 51°43′03.1″ N 5°10′24.8″ W); (**e**) *Spartina (Poaceae)* (12°22′4.0″ N 99°59′6.7″ E) (**f**) and *Suaeda* (Amaranthaceae) (12°10′19.6″ N 99°58′20.3″ E) in tidal marsh areas in southern Thailand.

**Figure 2:**
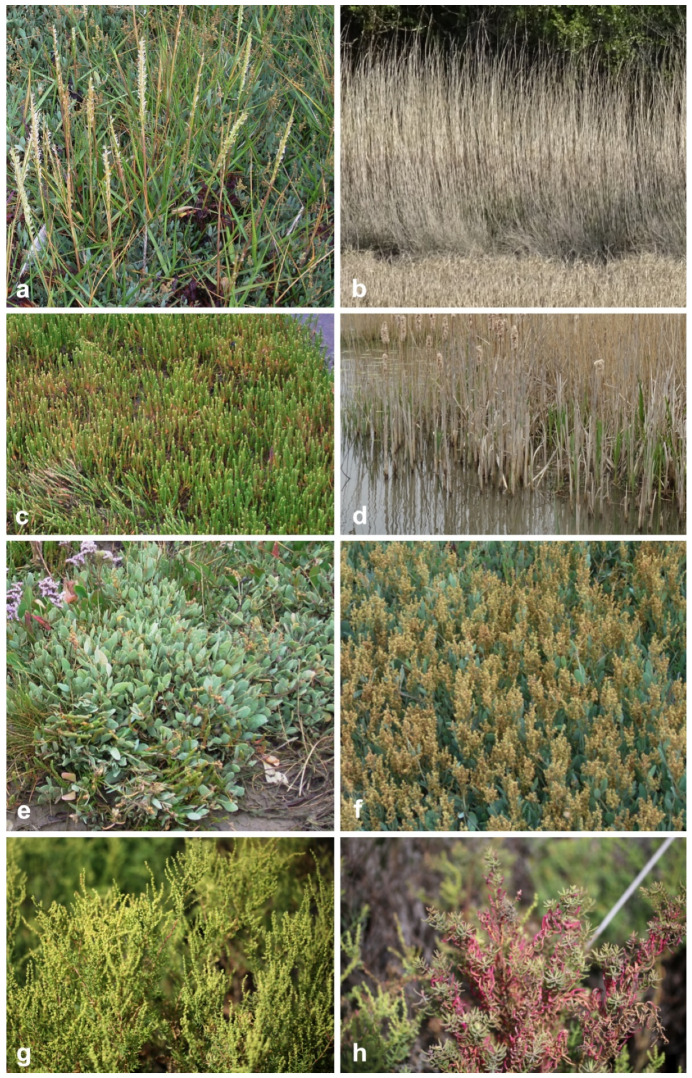
Halophytes in salt marsh ecosystems: (**a**) flowering inflorescence of *Spartina*, (**b**) *Phragmites*, (**c**) *Salicornia*, (**d**) *Typha*, (**e**,**f**) *Atriplex*, and (**g**,**h**) *Suaeda*.

There are over 500 species of salt marsh plants worldwide [9]. The families Amaranthaceae (subfamilies Chenopodiaceae, Salicornieae), Poaceae, Juncaceae, and Cyperaceae are the major vegetation in salt marsh ecosystems, while the minor components are Plumbaginaceae and Frankeniaceae [3], and are represented in Figure 1 and Figure 2. Salinity, latitude, region of the world, the frequency and duration of tidal flooding, substrate, oxygen and nutrient availability, surface elevation, competition among species, disturbance by wrack deposition are interacting factors that influence the species of halophytes in the salt marshes [10,11]. For example, *Spartina alterniflora* is a dominant grass from mid-tide to high-tide levels in temperate Eastern North America, while *Puccinellia* dominates in boreal and arctic marshes [10,11].

Major studies on halophytes focus on ecology and conservation [12,13,14]. One of these is the decomposition of vascular plant material wherein the detritus breakdown was reviewed in Pomeroy and Wiegert [15], Howarth and Hobbie [16], and Long and Mason [17]. The active decomposition processes in salt marsh ecosystems reflects to the relatively high rates of primary production. Three phases of plant decomposition were noted by Valiela et al. [18]. The early phase involves the leaching of soluble compounds, resulting in a fast rate of weight loss lasting for less than a month. Organic matter breakdown by microorganisms and continuous leaching of decayed products occurs in the second phase that lasts for a year. The last phase lasts for another year when there is a slow decay of refractory materials such as humates and fulvates [19].

The continuous breakdown of detritus into smaller fragments increases the surface-to-volume ratio and this is exposed to further microbial degradation. Bacteria and fungi are key decomposers in the salt marsh ecosystem that are essential for the transformation and recycling of nutrients through the environment. The colonization of fungi on standing dead halophytes commences during the early stages of decomposition before leaf fall to the salt marsh sediment surface [20,21]. The decomposition of the senescent tissues of halophytes by salt marsh fungi is brought about by the direct penetration of the host cell wall and the production of enzymes active in degrading lignocellulosic compounds, such as lignin, cellulose, and hemicellulose [22,23,24,25,26]. Bacterial communities are the major decomposers in the latter stage of decomposition [27,28]. Studies in salt marsh ecosystems not only consider microbial activity and the recycling of nutrients, but also bacterial [29,30] and fungal diversity [20,31,32].

The present review compiles the published data of fungi from halophytes, including their geographical distribution and host association. When compared to other fungal groups, salt marsh fungi are underexplored, and this review brings together the research efforts on these undiscovered habitats and plants. The pertinent literature from bibliographic databases (e.g., Scopus, Web of Science, Google Scholar) and published resources on salt marsh fungi documenting halophytes were compiled. Published works, wherein the documented fungal taxa were observed directly from halophytic substrates, are included (Table 1). The different host parts, living and dead, that are either partly or wholly submerged are documented, as well as drift plant portions washed up in salt marsh areas. Salt marsh fungi isolated using cultivation-dependent techniques were not included since it is not known if these fungi were actively growing and reproducing on the halophytes. The taxa were listed based on the recent outline of fungi and fungus-like taxa by Wijayawardene et al. [33]. Since previous works only listed the taxa and the hosts [34,35,36], here we include the plant parts where the fungus was observed, the location (country: state/province) where the host was collected, the life mode of the fungus, and the pertinent literature citations are included (Table 1). The accepted name of the host was based on the webpage of the World Flora Online consortium (http://www.worldfloraonline.org/; accessed on 10 May 2021), GrassBase (https://www.kew.org/data/grasses-db/sppindex.htm; accessed on 10 May 2021) and CRC World Dictionary of Grasses by Quattrocchi [37]. The graphs presented in the next sections summarizes the information from Table 1 and was developed using data visualization tools (Excel Office 365, Tableau Desktop Professional Edition 19.2.2).

## 2. Taxonomic Classification of Salt Marsh Fungi

### 2.1. Phyla

**Figure 3:**
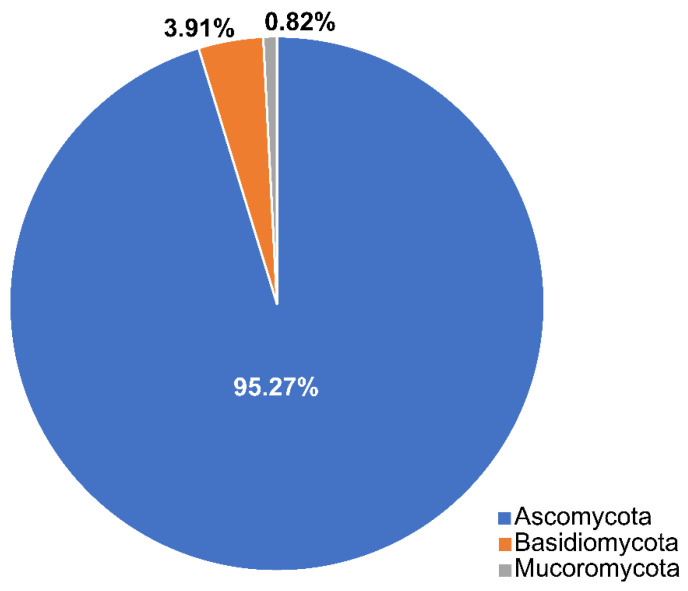
The distribution of salt marsh fungi among three fungal phyla.

**Figure 4:**
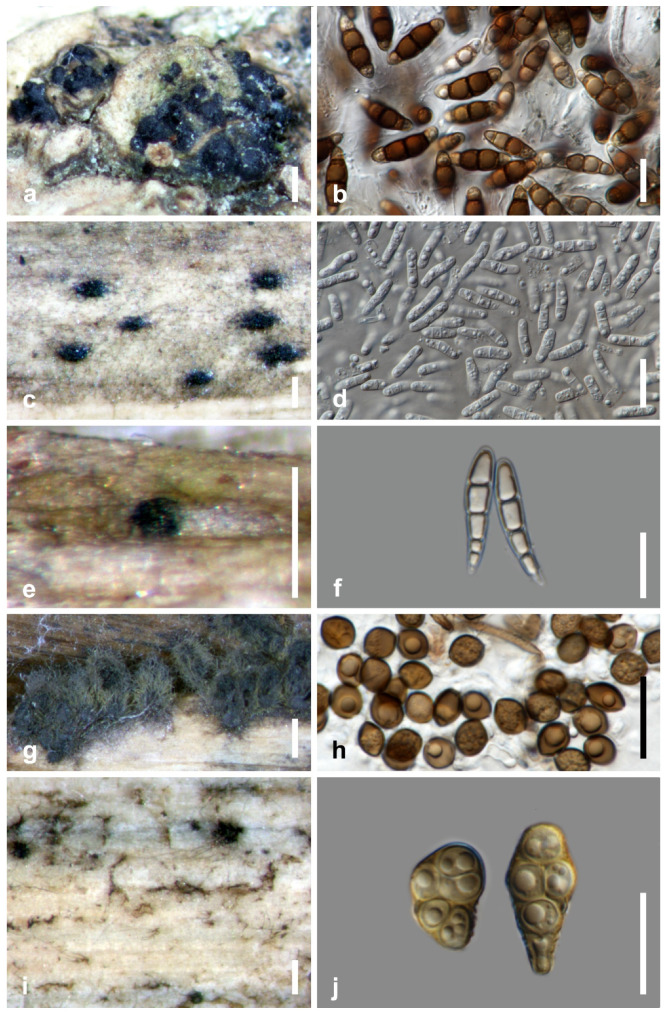
Salt marsh fungi. (**a**,**b**) *Halobyssothecium obiones* from *Atriplex portulacoides*; (**c**,**d**) *Halobyssothecium phragmites* from culms of *Phragmites* sp.; (**e**,**f**) *Buergenerula spartinae* from culms of *Spartina* sp.; (**g**,**h**) *Chaetomium* sp. from stem of *Typha* sp.; (**i**,**j**) *Alternaria* sp. from culms of *Spartina* sp. Scale bars: (**a**,**g**) = 500 µm; (**b**,**d**,**f**,**h**,**j**) = 20 µm; (**c**,**i**) = 200 µm; (**e**) = 100 µm.

Calado and Barata [34] documented 332 taxa associated with *Juncus roemerianus*, *Phragmites australis*, and *Spartina* spp. In this review, we list 486 taxa that belong to three phyla (Ascomycota, Basidiomycota, Mucoromoycota) (Table 1, Figure 3) and selected species are illustrated in Figure 4. Ascomycota dominates the taxa from salt marsh ecosystems, accounting for 95.27% (463 taxa). Nineteen species in twelve genera (*Aecidium*, *Chaetospermum*, *Falmingomyces*, *Merismodes*, *Nia*, *Parvulago*, *Puccinia*, *Sporobolomyces*, *Stilbum*, *Tranzscheliella*, *Tremella*, *Uromyces*) belong to Basidiomycota (3.91%), while Mucoromycota account for 0.82% (four species) of the salt marsh fungi.

### 2.2. Class

**Figure 5:**
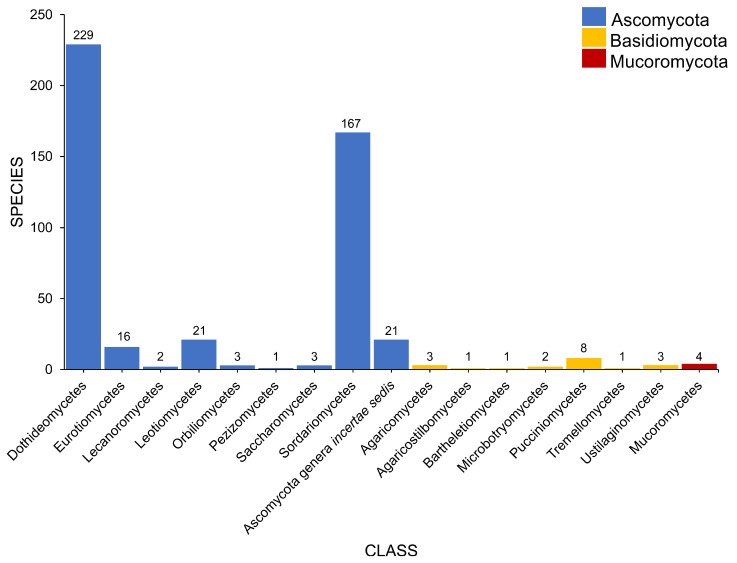
The distribution of salt marsh fungi in different fungal classes.

Salt marsh fungi are distributed into 17 classes (Table 1, Figure 5). Dothideomycetes has the highest number of taxa, which comprises 47.12% (229 taxa), followed by Sordariomycetes with 167 taxa (34.36%). Twenty-one species (in 20 genera) can be referred to as Ascomycota genera *incertae sedis*. The Ascomycetes with the least number of species include Leotiomycetes (21 species, 4.32%), Eurotiomycetes (16 species, 3.29%), Orbiliomycetes (3 species, 0.62%), Saccharomycetes (3 species, 0.62%), Lecanoromycetes (2 species, 0.41%), and Pezizomycetes (1 species, 0.21%).

Seven classes represent the Basidiomycota (Figure 5). Puccinomycetes has the highest number of taxa documented (eight species, three genera) followed by Agaricomycetes (three species, two genera), Ustilaginomycetes (three species, three genera), and Microbotryomycetes (two taxa, one genus). Agaricostilbomycetes, Bartheletiomycetes, and Tremellomycetes have one representative taxon each.

The Mucoromoycota account for the taxa *Blakeslea trispora*, *Mucor* sp., *Rhizopus stolonifera*, and *Syncephalastrum racemosum* [43,48,49].

### 2.3. Orders

**Figure 6:**
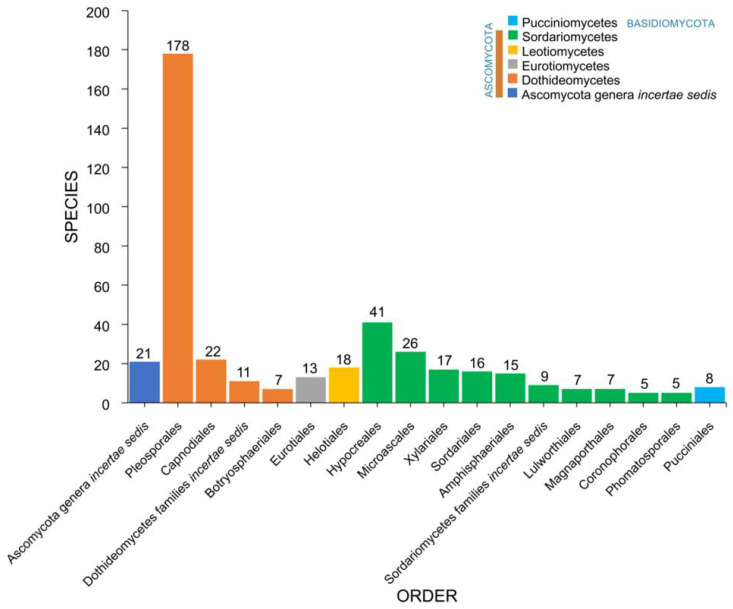
The distribution of salt marsh fungi in major fungal orders.

Salt marsh fungi recorded from different halophytes were distributed among 48 orders (Table 1, Figure 6). The Pleosporales is the largest order, with 178 taxa recorded followed by Hypocreales (41), Microascales (26), Capnodiales (22), Helotiales (18), Xylariales (17), Sordariales (16), Amphisphaeriales (15), and Eurotiales (13). The remaining 41 orders have less than 10 species (Table 1, Figure 5). Forty-two taxa belong to *incertae sedis* (Ascomycota genera *incertae sedis*: 21; Dothideomycetes families *incertae* sedis: 11; Sordariomycetes families *incertae sedis*: 9; Xylariomycetidae family *incertae sedis:* 1).

### 2.4. Families

**Figure 7:**
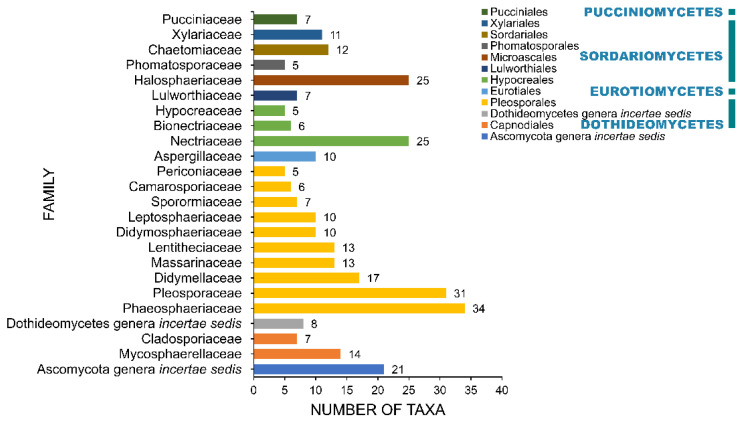
The distribution of salt marsh fungi among major fungal families.

A total of 108 families and 12 *incertae sedis* were recorded to be associated with salt marsh fungi (Table 1, Figure 7). Phaeosphaeriaceae and Pleosporaceae account for the largest families with 34 and 31 taxa recorded, respectively. Thirteen families have ten or more than taxa and include Nectriaceae (25), Halosphaeriaceae (25), Didymellaceae (17), Mycosphaerellaceae (14), Lentitheciaceae (13), Massarinaceae (13), Chaetomiaceae (12), Xylariaceae (11), Didymosphaeriaceae (10), Leptosphaeriaceae (10), and Aspergillaceae (10). The remaining 95 families have less than ten species recorded. Forty-four taxa are placed as *incertae sedis*, wherein 21 of these belong to Ascomycota genera *incertae sedis*.

## 3. Diversity of Fungi in Halophytes

**Figure 8:**
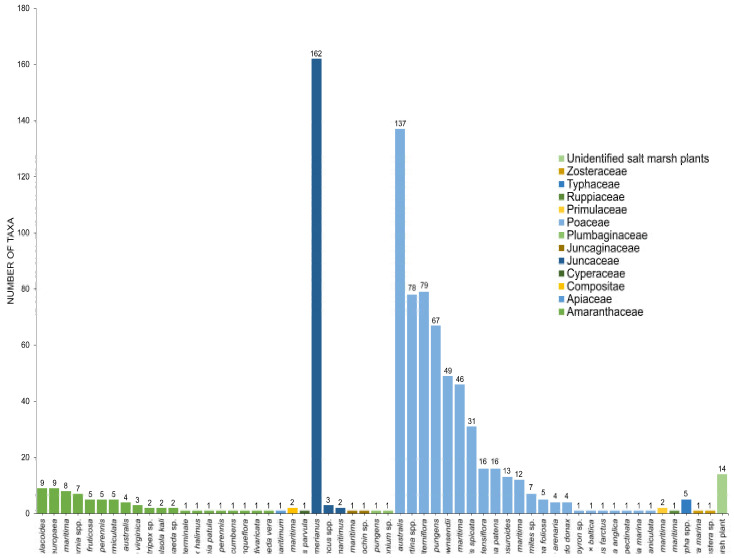
The number of taxa observed from different hosts in salt marsh ecosystems.

Twenty-seven genera under 11 families (Amaranthaceae, Apiaceae, Caryophyllaceae, Compositae, Juncaceae, Juncaginaceae, Plumbaginaceae, Poaceae, Poaceae, Primulaceae, Ruppiaceae, Typhaceae, Zosteraceae) of halophytes were reviewed for its fungal associates (Table 1, Figure 8). Halophytic species are represented in Figure 1 and Figure 2.

### 3.1. Amaranthaceae

**Figure 9:**
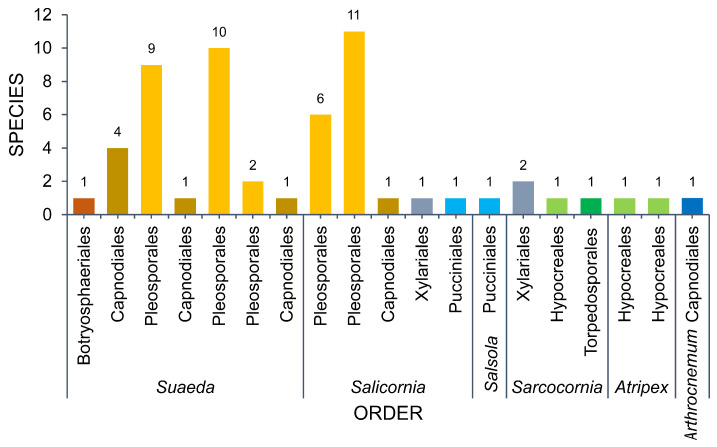
The number of taxa observed from Amaranthaceae.

Six genera (*Arthrocnemum*, *Atriplex*, *Salicornia*, *Salsola*, *Sarcocornia*, *Suaeda*) represent the Amaranthaceae. *Suaeda* and *Salicornia* are the most studied hosts in Amaranthaceae. Ascomycota account for 96.30% of the 52 taxa recorded in Amaranthaceae (Figure 9, Table 1). Two Pucciniomycetes species, *Aecidium suaedae* [154] and *Uromyces salicorniae* [95], represent Basidiomycota. The taxa in Amaranthaceae represent three classes wherein *Dothideomycetes* accounts for 85.19% (46 taxa), followed by *Sordariomycetes* with six taxa reported.

Fungi associated with *Suaeda* total 18 taxa. *Dothideomycetes* was represented by 14 taxa (77.78%), while three taxa were Sordariomycetes (*Cryptovalsa suaedicola* [144], *Fusarium fujikuroi* [62], *Moheitospora fruticosae* [130]) and one taxon of *Pucciniomycetes* (*Aecidium suaedae* [154]).

A total of 14 taxa were documented in *Salicornia*. Eleven of these belong to Dothideomycetes (Pleosporales: 10; Capnodiales: 1), followed by Sordariomycetes (two taxa: *Halocryptovalsa salicorniae* [145], *Tubercularia pulverulenta* [35]), and Pucciniomycetes (one taxon: *Uromyces salicorniae* [95]).

Fungi from *Atriplex* total 11 taxa (10 genera) and all of these belong to Pleosporales (Dothideomycetes). *Sarcocornia* harbors seven taxa (six Dothideomycetes, one Sordariomycetes). Only two taxa (*Alternaria* spp., *Stemphylium* spp.) and a single taxon (*Mycosphaerella salicorniae*) were reported from *Salsola* [35] and *Arthrocnemum* [35], respectively.

### 3.2. Poaceae

The association of fungi with grasses have been documented and most of the host plants are members of Poaceae. Ten genera of salt marsh grasses under Poaceae are included in this review wherein *Spartina* is the most studied of halophytic hosts for direct observation of marine fungi. In addition to *Spartina*, salt marsh grasses such as *Phragmites* and *Distichlis* were well studied also for their fungal associates.

Salt marsh fungi are not well-documented from grasses such as *Spartina anglica*, *S. pectinata*, *Spergularia marina*, *Uniola paniculata*, *Elymus farctus*, *× Ammocalamagrostis baltica*, and *Agropyron* sp. with one taxon recorded for each host [35]. Furthermore, there are few studies on the fungal composition of *Arundo donax* (4 taxa) [35] and *Ammophila arenaria* (four taxa). Marram grass (*Ammophila arenaria*) is more common in sand dunes and supports quite a diverse fungal community [157,158], while arbuscular mycorrhizal fungi (AMF) play a key role in the establishment, growth, and survival of plants [159].

#### 3.2.1. *Distichlis spicata*

Ascomycota dominates the taxa associated with *Distichlis spicata* (93.55%) wherein 16 and 13 species are members of Dothideomycetes and Sordariomycetes, respectively. Pleosporalean taxa constitute the majority of fungi associated with *D. spicata* (14 species), followed by Hypocreales with nine species recorded. *Puccinia distichlidis* and *Tranzscheliella distichlidis* represent the Basidiomycota. A total of 26 genera were recorded as associates of *D. spicata* and were mostly observed on senescent and decaying leaves.

#### 3.2.2. *Elymus pungens*

**Figure 10:**
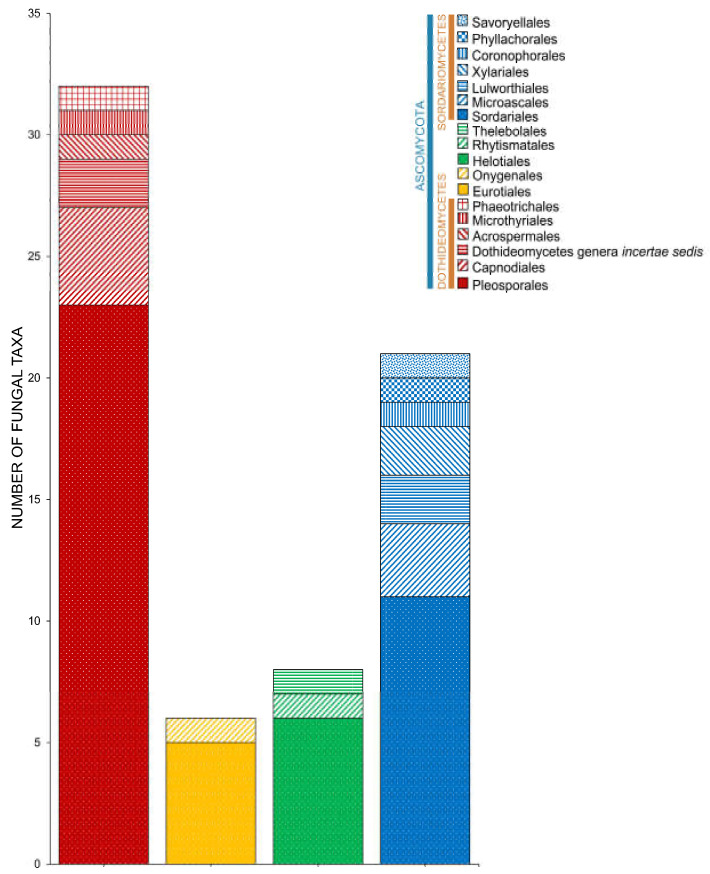
The distribution of fungal taxa associated with *Elymus pungens*.

Sixty-seven taxa were recorded in *Elymus pungens* and belong to Ascomycota. Most of the taxa belong to Dothideomycetes (32 taxa), followed by Sordariomycetes (21 taxa), Leotiomycetes, and Eurotiomycetes (6 taxa) (Table 1, Figure 10).

#### 3.2.3. *Puccinellia maritima*

A total of 12 taxa (six Sordariomycetes; the following five Dothideomycetes: *Micronectriella agropyri*, *Lautitia danica*, *Leptosphaeria pelagica*, *Septoriella vagans*, *Paradendryphiella salina*; one Leotiomycetes: *Thelebolus crustaceus*) were recorded in *Puccinellia maritima* [38]. All the taxa from Sordariomycetes belong to Sordariales (*Chaetomium elatum*, *C. globosum*, *C. thermophilum*, *Corynascus sepedonium*, *Thermothielavioides terrestris*, *Sordaria fimicola*) [38].

#### 3.2.4. *Spartina*

**Figure 11:**
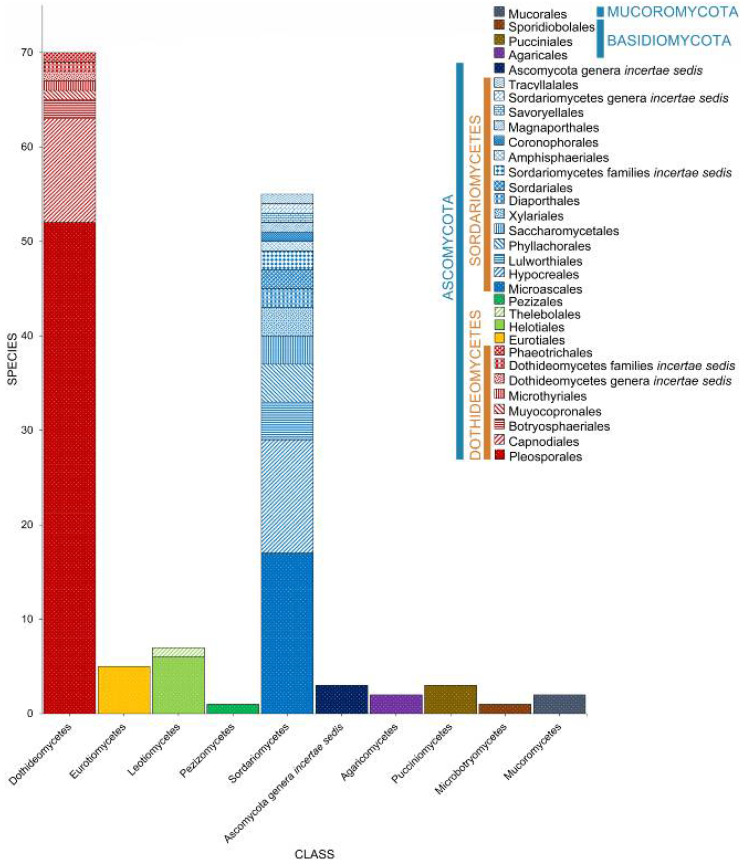
The distribution of fungal taxa associated with *Spartina*.

A total of 149 taxa (141 Ascomycota, 6 Basidiomycota, 2 Mucoromycota) were recorded in *Spartina*. The majority of the taxa belong to Dothideomycetes (70 taxa), followed by Sordariomycetes (59 taxa). Pleosporaceae and Halosphaeriaceae dominate the fungi documented in *Spartina* with 19 and 17 taxa recorded, respectively. *Spartina alterniflora*, *S. maritima*, and *Spartina × townsendii* harbor 79, 46, and 49 taxa, respectively (Figure 11, Table 1). A total of 78 taxa were recorded in the unidentified *Spartina* species. The identification of the *Spartina* species can be challenging, wherein species are morphologically similar.

*Halobyssothecium obiones* was recorded from six species of *Spartina* (*S. alterniflora* [20,35,52,61,71,74,80,81,82], *S. cynosuroides* [35], *S. densiflora* [64], *S. maritima* [31,54,59,63], *S. patens* [36], *S. townsendii* [49,65], and the unidentified *Spartina* sp. [32,35,36,58,84]), while six *Spartina* spp. harbors unidentified *Mycosphaerella* species. Six species (*Leptosphaeria pelagica*, *Lulworthia* spp., *Phaeosphaeria halima*, *Phaeosphaeria spartinicola*, *Phoma* spp., *Stagonospora* spp.) were recorded in five different hosts. The unidentified *Spartina* species harbors 28 unique species. Amongst the taxa found in *Spartina*, 32 species can only be found in *S. alterniflora*, while *S. maritima* harbors 21 unique species, the most intensively surveyed species.

#### 3.2.5. *Phragmites*

**Figure 12:**
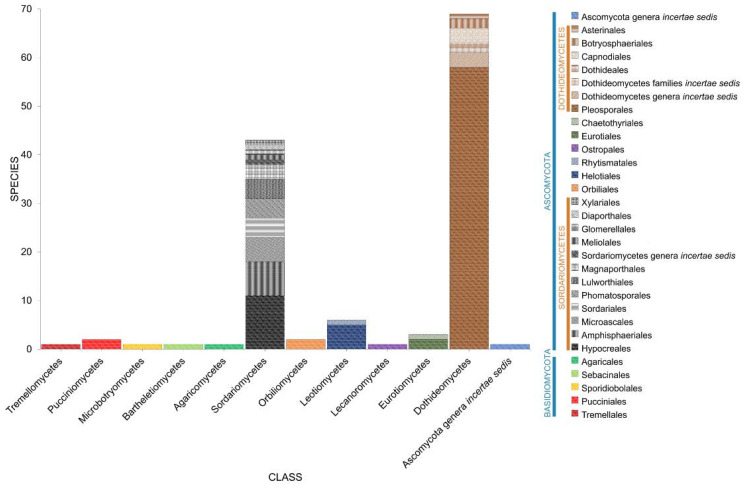
The distribution of fungal taxa associated with *Phragmites*.

A total of 138 taxa have been documented in *Phragmites* (Figure 12, Table 1). Most of the taxa belong to Ascomycota (131 taxa), while six taxa represent the Basidiomycota. Dothideomycetes dominates half of the taxa in *Phragmites* (71 taxa, 51.45%) followed by Sordariomycetes (44 taxa, 31.88%), Leotiomycetes (6 taxa, 4.35%), Ascomycota genera *incertae sedis* (5 taxa, 3.62%), Eurotiomycetes (3 taxa, 2.17%), Orbiliomycetes (2 taxon, 1.45%), and Pucciniomycetes (1 taxa, 1.45%). One taxon each were recorded to Agaricomycetes [40], Bartheletiomycetes [41], Lecanoromycetes [39], Microbotryomycetes [39,50], and Tremellomycetes [39,40]. Pleosporalean taxa accounts for the highest number of fungi associated with *Phragmites* (42.75%, 59 taxa).

*Phragmites australis* harbors diverse fungi that totals to 137 taxa (101 genera) [39,40,41,50,79,115]. Seven species (*Arthrinium arundinis* [62], *Halazoon fuscus* [87], *Halobyssothecium phragmitis* [85], *Keissleriella linearis* [85], *Phomatospora dinemasporium* [62], *Remispora hamata* [87], *Setoseptoria phragmitis* [87]) were recorded in unidentified *Phragmites* species.

### 3.3. Juncaceae

**Figure 13:**
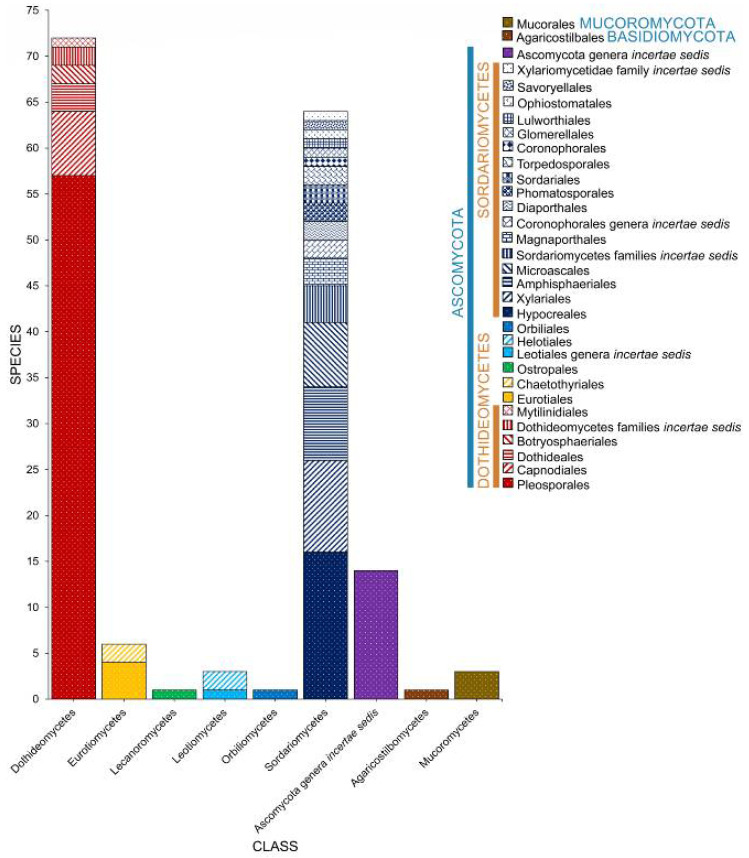
The distribution of fungal taxa associated with *Juncus*.

*Juncus roemerianus*, *J. maritimus*, and an unidentified *Juncus* species represent Juncaceae. Salt marsh fungi are diverse in *Juncus* and dominated by Ascomycota, which constitutes 97.58% of the 165 reported taxa (Figure 13, Table 1). *Stilbum* sp. represented the Basidiomycota, while three taxa (*Blakeslea trispora*, *Mucor* sp., *Syncephalastrum racemosum*) of Mucoromycota were recorded. Dothideomycetes and Sordariomycetes account for the highest number of *Juncus*-associated fungi with 72 (43.64%) and 64 (38.79%) taxa documented.

*Juncus roemerianus* has been extensively studied for its associates with 162 documented taxa [32,42,43,60,66,76,77,78,97,98,104,105,110,116,117,118,135,147,148]. Few species were reported to *Juncus maritimus* that harbor only two taxa (*Leptosphaeria albopunctata*, *Phaeosphaeria neomaritima*) [35]. *Phaeosphaeria neomaritima* [36,52,71,80], *P. spartinicola* [52], and *Monodictys pelagica* [35] were observed in an unidentified species of *Juncus*.

*Phragmites australis* harbors diverse fungi that totals to 137 taxa (101 genera) [39,40,41,50,79,115]. Seven species (*Arthrinium arundinis* [62], *Halazoon fuscus* [87], *Halobyssothecium phragmitis* [85], *Keissleriella linearis* [85], *Phomatospora dinemasporium* [62], *Remispora hamata* [87], *Setoseptoria phragmitis* [87]) were recorded in unidentified *Phragmites* species.

### 3.4. Other Families

Few reports on salt marsh fungi are from the following hosts: Apiaceae: *Crithmum maritimum* (one taxon: *Phoma* sp.), Typhaceae: *Typha* spp. (five taxa: *Arundellina typhae*, *Chaetomium* sp., *Magnisphaera spartinae*, *Pleospora pelagica*, *Remispora hamata*); Compositae: *Artemisia maritima* (two taxon: *Neocamarosporium artemisiae*, *N. maritimae*); Caryophyllaceae: *Spergularia marina* (one taxon: *Cladosporium algarum*); Plumbaginaceae: *Limonium* sp. (one taxon: *Mycosphaerella salicorniae*); *Armeria pungens* (one taxon: *Mycosphaerella staticicola*); Juncaginaceae: *Triglochin* sp. and *T. maritima* (one taxon: *Stemphylium triglochinicola*); Primulaceae: *Lysimachia maritima* (two taxa: *Leptosphaeria orae-maris*, *Stemphylium vesicarium*); Ruppiaceae: *Ruppia maritima* (one taxon: *Flamingomyces ruppiae*); and Zosteraceae: *Zostera marina* (one taxon: *Corollospora ramulosa*) and *Zostera* sp. (*Asteromyces cruciatus*). Alva et al. [160] report *Penicillium chrysogenum* as an endophyte from *Zostera japonica*.

Fourteen taxa were documented from unidentified salt marsh plants. All of the taxa belong to Ascomycota (seven Dothideomycetes, five Sordariomycetes, one Eurotiomycetes). Pleosporalean taxa from six families account for half of the taxa (the following seven species: *Camarosporium palliatum*, *C. roumeguerei*, *Coniothyrium obiones*, *Halobyssothecium obiones*, *Periconia* sp., *Loratospora aestuarii*, *Pleospora pelvetiae*).

## 4. Geographical Distribution of Salt Marsh Fungi

**Figure 14:**
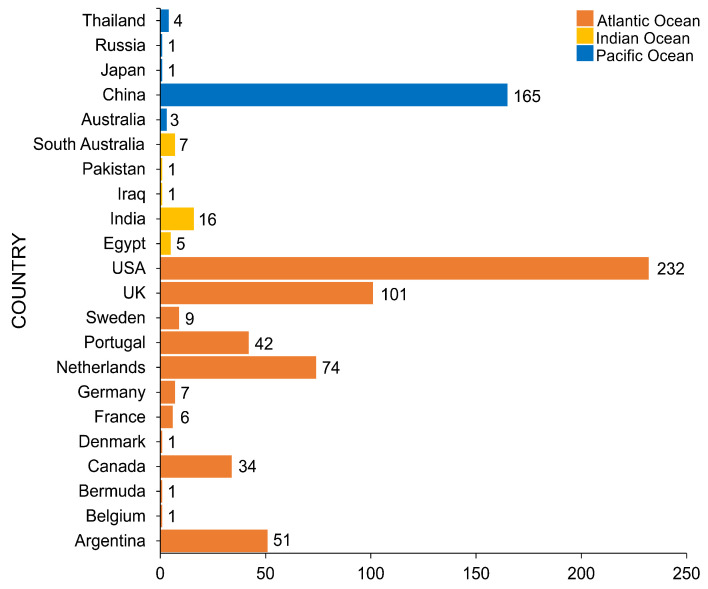
The number of salt marsh fungi reported in the Pacific, Atlantic, and Indian Oceans.

The salt marsh fungi reported are from countries of three major oceans, as documented in Figure 14. The Atlantic Ocean consists of 12 countries, wherein the USA had the highest number of species recorded (232 taxa) followed by the UK (101 taxa), the Netherlands (74 taxa), and Argentina (51 taxa). China had the highest number of salt marsh fungi in the Pacific Ocean with 165 taxa reported, while in the Indian Ocean, India reported the highest taxa (16 taxa). Most of the biodiversity studies documenting salt marsh fungi in the Atlantic Ocean are mostly from the USA and the UK and this reflects the high number of taxa [32,36,38,49,61]. China ranked second with the most number of salt marsh fungal taxa, mainly due to the biodiversity study in *Phragmites australis* conducted by Poon et al. [41].

**Figure 15:**
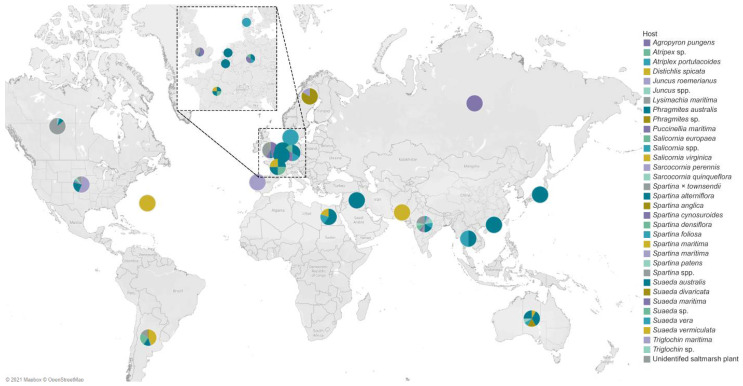
Map of countries showing the global distribution of fungal diversity studies in halophytes. The different color of each pie chart represents the hosts, and the angle measured the number of their fungal associates.

The geographical distribution of salt marsh fungi and the different halophytes are presented in Figure 15. The fungi associated with salt marsh grass *Phragmites australis* have been studied in different countries (Australia, Belgium, Egypt, France, Germany, China, Iraq, Japan, the Netherlands, South Australia, Thailand). *Spartina alterniflora* was recorded in countries along the Atlantic (Argentina, Canada, France, USA) and the Indian Ocean (India), but lacks data from countries in the Pacific Ocean.

### United States of America

**Figure 16:**
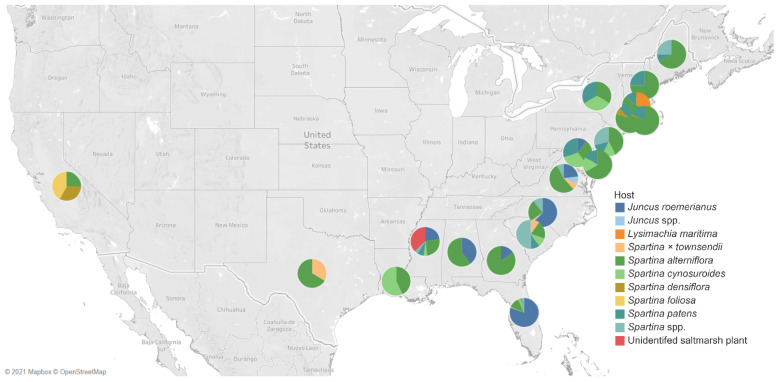
Map of the United States of America (USA) showing the distribution of fungal diversity studies of halophytes in different states. The different color of each pie chart represents the hosts, and the angle measured the number of their fungal associates.

Most of the studies of halophytes-associated fungi were concentrated on the United States of America (USA) (Figure 16). Table 1 lists the salt marsh fungi in 20 states. Florida has been the frequently studied, wherein seven hosts (*Juncus roemerianus*: 108 taxa; *Spartina × townsendii:* 1; *Spartina alterniflora*: 16; *Spartina cynosuroides*: 3; *Spartina densiflora*: 1; *Spartina patens*: 2; *Spartina* spp.: 3) were observed for salt marsh fungi. Six hosts were studied in North Carolina, wherein *Juncus roemerianus* harbored the highest number of fungi (48 taxa). In Rhode Island, *Spartina alterniflora* accounts for the highest number of fungi, with 41 taxa recorded.

## 5. Conclusions and Future Perspectives

Most studies of fungi on salt marsh plants are from *Spartina*, *Juncus*, and *Phragmites*, probably due to the huge biomass generated by these taxa. The mycota of less bulky halophytes (e.g., *Limonium*, *Triglochin*, *Uniola*) and litter from the surrounding sea grass beds washed off to marsh areas (e.g., *Zostera japonica*, *Z. marina*, *Z. noltii*) are also less represented, or these hosts are yet to be explored. The checklist presented in the current study updates the list of Calado and Barata [34] and the inclusion of fungi associated with rarely studied halophytes record 486 taxa worldwide. Ascomycota dominate the taxa (463 taxa) and are comprised mostly of Dothideomycetes with their ability to eject their ascospores forcibly and widely, spore type, the formation of ascomata or ascostromata under a clypeus or just immersed in thin leaves, and an ability to decompose lignocellulose substrates [57,161]. Meyers et al. [162] showed that salt marsh yeasts and the ascomycete, *Buergenerula spartinae*, produce degradative enzymes and utilize simple carbon and nitrogen compounds. The yeast, *Pichia spartinae*, produces β-glucosidase and other degradative enzymes. Gessner [74] demonstrated that a number of salt marsh fungi isolated from *Spartina alterniflora*, *Zostera* sp., and *Z. marina* produced enzymes capable of degrading cellulose, cellobiose, lipids, pectin, starch, tannic acid, and xylan and, thus, play a key role in the degradation of storage and structural compounds. Salt marsh fungi might possess high biotransformation and metabolic abilities, which could be related to their ecology.

Basidiomycota (19 taxa) and Mucoromycota (4 taxa) are poorly represented in salt marsh ecosystems as they are in other marine habitats [163]. There are no records of Chytridiomycota listed in the present work and only a few authors detected this group, and other basal fungal lineages, in salt marsh ecosystems using molecular analysis [164,165,166,167]. These groups are worth exploring to determine the overall fungal communities in the salt marsh ecosystems. Many chytrids and other basal fungi are more challenging to cultivate and require different isolation methods (e.g., baiting techniques in liquid culture) than the saprobes, methods that have rarely been applied in the study of saltmarsh plants. When appropriate techniques are used, chytrids and other zoosporic organisms have been reported. For example, the fungal-like organism *Phytophthora inundata* has been recovered from the halophilic plants *Aster tripolium* and *Salicornia europaea*, while *P. gemini* and *P. chesapeakensis* occur on *Zostera marina*, and *Salisapilia nakagirii* on the decaying litter of *Spartina alterniflora* (www.marinefungi.org; accessed on 10 May 2021, [163]). Marine chytrids have been isolated from substrates such as seaweeds and mangrove leaves [163].

The taxa listed are mostly saprobes and these can be attributed to the inclusion of salt marsh fungi observed directly from the different host parts, which are mostly submerged decaying substrates. When compared to saprobic fungi in halophytes, few studies have been carried out on the diversity of endophytes and pathogens and their interaction in the salt marsh ecosystems. Surveys on endophytic fungi from halophytes using cultivation-dependent methods coupled with molecular approaches, showed that endophytes were dominated by Ascomycota and a few belonged to Basidiomycota and Zygomycota [168,169,170,171,172,173,174,175]. Pathogenic fungi from salt marsh ecosystems are poorly documented but play a significant role in the dynamics of the ecosystem [176,177,178]. For example, Govers et al. [179] reported that the fungal-like organisms *Phytophthora gemini* and *P. inundata* caused widespread infection of the common seagrass species, *Zostera marina* (eelgrass), across the northern Atlantic and Mediterranean that threatened the conservation and restoration of vegetated marine coastal systems. Likewise, *Claviceps purpurea* affects the viability of *Spartina townsedii* in south coast UK salt marshes. Fisher et al. [180] noted that *Cl. purpea* in the Alabama and Mississippi coastlines rendered the seeds of one of the primary salt marsh grasses sterile. Raybold et al. [181] recorded epidemics of *C. purpurea* on *Spartina anglica* in Poole Harbor (UK) and that ergot growth was detrimental to seed production. These underexplored fungal groups are worthy to be explored for their ecological and biotechnological importance.

This shows how salt marsh fungal studies were concentrated in countries in the Atlantic Ocean specifically the USA (232 taxa) and the UK (101 taxa). Many salt marsh areas remain unexplored, especially those in the Indian and Pacific Oceans, and these areas are hotspots of biodiversity and novel fungal taxa based on the exploration of various habitats [85,100,163,182,183,184,185,186,187]. Recently, novel species were isolated in halophytes [85,100,145] and further taxa remain to be discovered, isolated, and sequenced, while vast areas worldwide have yet to be surveyed. For example, salt marsh plants are immensely numerous, diverse, and common along the south-east coast of Australia, yet little is known of their fungal associates [188].

The salt marsh vegetation and its fungal associates are adapted to salt stress and inundation and are subjected to extreme environmental conditions such as being periodically wet to different lengths of time leading to drying out at low tides and exposure to high temperatures and drying out at midday. Many are well adapted to prevailing conditions by their fleshy leaves (*Suaeda australis*), others can tolerate high flooding.

**Figure 17:**
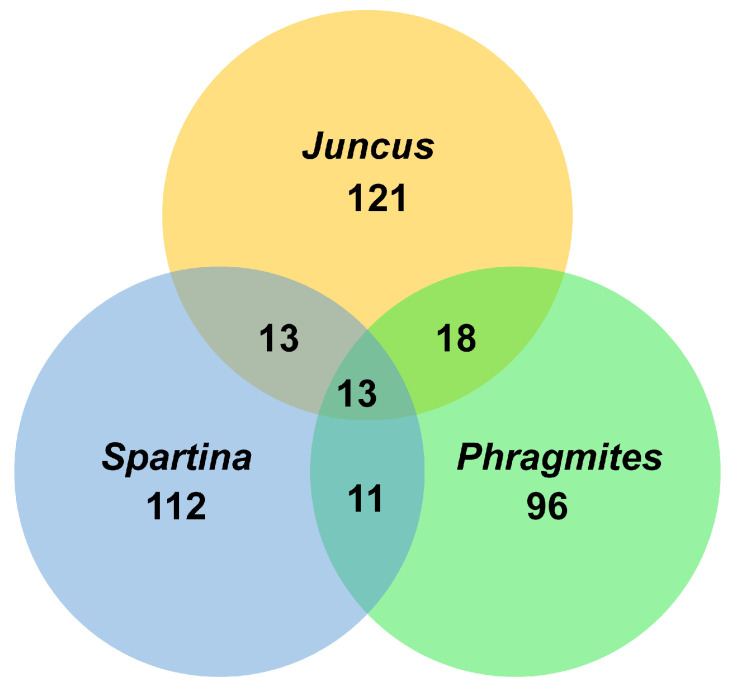
Venn diagram showing the association of salt marsh fungi from commonly studied halophytes.

Few data are currently available on the specificity of fungi on their salt marsh hosts. Figure 17 shows the number of fungal taxa recorded from the three commonly studied hosts, *Juncus*, *Phragmites*, and *Spartina*, wherein there is little overlap in the species composition. One of the common species on *Spartina* plants is undoubtedly *Halobyssothecium obiones*, while *Leptosphaeria pelagica* is common. A common ascomycete on *Atriplex portulacoides* and *Suaeda maritima* is *Decorospora gaudefroyi*. Host plants that have been little surveyed for fungi are *Limonium vulgare* (sea lavender) and *Atriplex portulacoides* (sea purslane), yet they do support a number of taxa, e.g., *Neocamarosporium obiones* and *Amarenomyces ammophilae*. The fungal community reported on *Juncus roemerianus* in the salt marsh at North Carolina is significantly different from those on *Spartina* and *Phragmites*. It remains to be seen if this is due to the host plant or its geographical location.

Another groups of fungi that have not been fully studied in the salt marsh habitat are yeasts, as these also require specific techniques for their isolation from the water column or from plant tissue. Spencer et al. [189] recovered a number of yeasts from the vicinity of *Spartina townsendii*, as follows: very numerous *Cryptococcus* spp.; *Trichosporon cutaneum*; *Trichosporon pullulans*; the relatively rare species, *Metschnikowia bicuspidata and Cryptococcus flavus;* and *Saturnospora ahearnii* [190]. Although marine yeasts are common in sea water and deep seawater vents [163], their large-scale sampling in salt marshes remains a challenge for the future.

Currently, the salt marsh ecosystem has been threatened both by global warming and human activity. Sea-level rises brought about by climate change alter the location and character of the land–sea interface wherein salt marsh vegetation moves upward and inland. The increase in the sea level may not lead to the loss of coastal marshes, but the resiliency will depend on the ability of halophytes to migrate upland. Susceptible areas are organogenic marshes and areas where sediment is limited, potentially leading to catastrophic shifts and marsh loss. In this paper, a total of 57 plant taxa under 27 genera were reviewed for their fungal associates. The halophytes included here are only approximately 11% of the total number of species of salt marsh plants worldwide. Thus, many salt marsh fungi await discovery with wider host plant sampling and the use of a wider range techniques for their isolation. For this reason, it is imperative to study the halophytic fungi to document not just biodiversity but also to discover novel taxa restricted only to this kind of habitat.

## Figures and Tables

**Table 1:** Geographical distribution of salt marsh fungi recorded from various halophytes.

Taxon	Host Part	Life Mode	Hosts	Distribution	References
**ASCOMYCOTA**					
**DOTHIDEOMYCETES**					
**Acrospermales**					
**Acrospermaceae**					
*Acrospermum graminum* Lib.	–	–	*Elymus pungens*	UK	[38]
**Asterinales**					
**Morenoinaceae**					
*Morenoina phragmitis* J.P. Ellis	Living/decomposing leaf sheaths and stems	Saprobic	*Phragmites australis*	Netherlands: Zeeland	[39,40]
**Botryosphaeriales**					
**Botryosphaeriaceae**					
*Botryosphaeria festucae* (Lib.) Arx and E. Müll.	Living/decomposing leaf sheaths and stems	Saprobic	*Phragmites australis*	Netherlands: Zeeland	[39,40]
*Macrophomina* sp.	Decaying stems and leaf sheaths	Saprobic	*Phragmites australis*	China: Hong Kong	[41]
*Tiarosporella halmyra* Kohlm. and Volkm.-Kohlm.	Senescent culms	Saprobic	*Juncus roemerianus*	USA: North Carolina	[42]
**Phyllostictaceae**					
*Guignardia* spp.	Senescent leaves	Saprobic	*Juncus roemerianus*	USA: Florida	[43]
*Phyllosticta* sp.	–	Pathogenic	*Spartina cynosuroides*	USA: Maryland	[44]
*Phyllosticta spartinae* Brunaud	–	–	*Spartina maritima*	France	[45]
*Phyllosticta suaedae* Lobik	Leaves	–	*Suaeda maritima*	Russia	[46]
**Capnodiales**					
**Cladosporiaceae**					
*Cladosporium algarum* Cooke and Massee	–	–	*Spergularia marina*	–	[35]
	–	–	*Suaeda maritima*	–	[35]
*Cladosporium allicinum* (Fr.) Bensch, U. Braun and Crous	–	–	*Elymus pungens*	UK	[38]
*Cladosporium cladosporioides* (Fresen.) G.A. de Vries	Leaves	Saprobic	*Distichlis spicata*	Argentina: Buenos Aires	[47]
	Living, senescent, and decaying leaves	Saprobic	*Juncus roemerianus*	USA: Florida	[43]
	Leaves and roots	Saprobic	*Spartina* sp.	Canada: Bay of Fundy	[48]
*Cladosporium herbarum* (Pers.) Link	Leaves	Saprobic	*Distichlis spicata*	Argentina: Buenos Aires	[47]
	Stem	Saprobic	*Spartina townsendii*	UK: England	[49]
	Leaves, stems, and roots	Saprobic	*Spartina* sp.	Canada: Bay of Fundy	[48]
*Cladosporium macrocarpum* Preuss	Leaves	Saprobic	*Spartina* sp.	Canada: Bay of Fundy	[48]
*Cladosporium sphaerospermum* Penz.	Living, senescent, and decaying leaves	Saprobic	*Juncus roemerianus*	USA: Florida	[43]
	Living/decomposing leaf sheaths and blades	Saprobic	*Phragmites australis*	Netherlands: Zeeland	[39,41,50]
	–	Saprobic	*Spartina patens*	USA: Rhode Island	[36]
	–	Saprobic	*Spartina* sp.	Canada	[36]
**Capnodiales genera *incertae sedis***					
*Mucomycosphaerella eurypotami* (Kohlm., Volkm.-Kohlm. and O.E. Erikss.) Quaedvl. and Crous	Senescent leaves	Saprobic	*Juncus roemerianus*	USA: North Carolina	[51]
**Mycosphaerellaceae**					
*Fulvia fulva* (Cooke) Cif.	Leaves and stems	Saprobic	*Spartina* sp.	Canada: Bay of Fundy	[48]
*Micronectriella agropyri* Apinis and Chesters	–	–	*Elymus pungens*	UK	[38]
	–	–	*Puccinellia maritima*	UK	[38]
	–	–	*Spartina townsedii*	UK	[38]
*Mycosphaerella lineolata* (Roberge ex Desm.) J. Schröt.	Living/decomposing leaf sheaths and stems	Saprobic	*Phragmites australis*	Netherlands: Zeeland	[39,40]
	–	–	*Elymus pungens*	UK	[38]
*Mycosphaerella salicorniae* (Auersw.) Lindau	–	–	*Arthrocnemum subterminale*	–	[35]
	–	–	*Limonium* sp.	–	[35]
	–	–	*Sarcocornia perennis*	–	[35]
	–	–	*Salicornia fruticosa*	–	[35]
	–	–	*Salicornia procumbens*	–	[35]
	–	–	*Salicornia europaea*	–	[35]
	–	–	*Salicornia perennis*	–	[35]
	–	–	*Sarcocornia fruticosa*	–	[35]
	Drying stalks and inflorescence	Saprobic	*Salicornia* sp.	India	[52]
	Dried inflorescences	Saprobic	*Salicornia virginica*	Bermuda	[35,53]
	–	Saprobic	*Spartina marítima*	Portugal: Alentejo, Lisbon	[54]
	–	–	*Suaeda vermiculata*	–	[35]
	Drying stalks and inflorescence	Saprobic	*Suaeda* sp.	India	[52]
*Mycosphaerella* spp.	–	–	*Elymus pungens*	UK	[38]
	Senescent and decaying leaves	Saprobic	*Juncus roemerianus*	USA: Florida, Mississippi	[43,55]
	Decaying leaves, leaf blades	Saprobic	*Spartina alterniflora*	Argentina: Buenos Aires; USA: Alabama, California, Georgia, Mississippi	[25,35,36,55,56,57,58]
	–	–	*Spartina* cf. *densiflora*	USA: California	[25,35]
	–	–	*Spartina* cf. *pectinata*	–	[35]
	–	–	*Spartina* sp.	Argentina: Buenos Aires; Canada	[35,36]
	Decaying leaf blades	Saprobic	*Spartina foliosa*	USA: California	[25]
	Leaf sheaths and blades, stem	Saprobic	*Spartina marítima*	Portugal: Alentejo, Lisbon, Centro	[54,59]
*Mycosphaerella staticicola* (Pat.) Dias	–	–	*Armeria pungens*	–	[35]
*Mycosphaerella suaedae-australis* Hansf.	–	–	*Suaeda australis*	–	[35]
*Rivilata ius* Kohlm., Volkm.-Kohlm. and O.E. Erikss.	Tips of senescent, very old, and brittle leaves	Saprobic	*Juncus roemerianus*	USA: North Carolina	[60]
*Septoria* spp.	Living, senescent, and decaying leaves	Saprobic	*Juncus roemerianus*	USA: Florida	[43]
	Living/decomposing leaf sheaths	Saprobic	*Phragmites australis*	Netherlands: Zeeland	[39]
	Upper leaves, inflorescence, seeds	Saprobic	*Spartina alterniflora*	USA: Rhode Island	[61]
*Septoria suaedae-australis* Hansf.	Dead stems	Saprobic	*Suaeda australis*	South Australia	[62]
*Sphaerulina albispiculata* Tubaki	Sheath	Saprobic	*Spartina marítima*	Portugal: Alentejo, Lisbon	[54]
	Stem	Saprobic	*Spartina marítima*	Portugal: Alentejo	[63]
*Sphaerulina orae-maris* Linder	–	–	*Ammophila arenaria*	–	[35]
	Rhizome and root	Saprobic	*Spartina densiflora*	Argentina: Buenos Aires	[64]
	Leaf sheaths and blades, stem	Saprobic	*Spartina marítima*	Portugal: Alentejo, Lisbon, Algarve, Centro	[31,54,59,63]
*Sphaerulina pedicellata* T.W. Johnson	–	Saprobic	*Spartina townsendii*	–	[65]
	Attached culms, stems	Saprobic, parasitic	*Spartina alterniflora*	USA: Rhode Island	[20,61]
*Sphaerulina* sp.	Senescent and decaying leaves	Saprobic	*Juncus roemerianus*	USA: Florida	[43]
**Dothideales**					
**Saccotheciaceae**					
*Aureobasidium* sp.	Living, senescent, and decaying leaves	Saprobic	*Juncus roemerianus*	USA: Florida	[43]
*Pseudoseptoria donacis* (Pass.) B. Sutton	Living/decomposing leaf blades and sheaths	Saprobic	*Phragmites australis*	Netherlands: Zeeland	[39,50]
*Selenophoma* sp.	Senescent and decaying leaves	Saprobic	*Juncus roemerianus*	USA: Florida	[43]
**Dothideaceae**					
*Scirrhia annulata* Kohlm., Volkm.-Kohlm. and O.E. Erikss.	Senescent culms and leaves	Saprobic	*Juncus roemerianus*	USA: North Carolina	[66]
**Dothideomycetes families *incertae sedis***					
**Eriomycetaceae**					
*Heleiosa barbatula* Kohlm., Volkm.-Kohlm. and O.E. Erikss.	Senescent leaves	Saprobic	*Juncus roemerianus*	USA: North Carolina	[66]
**Pseudorobillardaceae**					
*Pseudorobillarda phragmitis* (Cunnell) M. Morelet	Decaying stems and leaf sheaths	Saprobic	*Phragmites australis*	China: Hong Kong	[41,67]
*Pseudorobillarda* sp.	Dead stems	Saprobic	*Spartina alterniflora*	Canada	[36]
**Dothideomycetes genera *incertae sedis***					
*Bactrodesmium atrum* M.B. Ellis *Lautitia danica* (Berl.) S. Schatz	Living/decomposing stems	Saprobic	*Phragmites australis*	Netherlands: Zeeland	[40]
	–	–	*Elymus pungens*	UK	[38]
	–	–	*Puccinellia maritima*	UK	[38]
*Monodictys austrina* Tubaki	Senescent leaves	Saprobic	*Juncus roemerianus*	USA: Florida	[43]
*Monodictys castaneae* (Wallr.) S. Hughes	Leaves	Saprobic	*Spartina* sp.	Canada: Bay of Fundy	[48]
*Neottiosporina australiensis* B. Sutton and Alcorn	Living/decomposing leaf blades and sheaths, stems	Saprobic	*Phragmites australis*	Netherlands: Zeeland	[39,40,50]
*Neottiosporina* sp.	Decaying stems and leaf sheaths	Saprobic	*Phragmites australis*	China: Hong Kong	[41]
*Otthia* sp.	Senescent leaves	Saprobic	*Juncus roemerianus*	USA: Florida	[43]
*Trichometasphaeria setulosa*. (Sacc. and Roum.) Apinis and Chesters ined.	–	–	*Elymus pungens*	UK	[38]
*Trichometasphaeria* sp.	–	–	*Elymus pungens*	UK	[38]
Microthyriales					
Microthyriaceae					
*Microthyrium microscopicum Desm*.	–	–	*Spartina patens*	–	[68]
*Microthyrium gramineum* Sacc., E. Bommer and M. Rousseau	–	–	*Elymus pungens*	UK	[38]
**Muyocopronales**					
**Muyocopronaceae**					
*Ellisiodothis inquinans* (Ellis and Everh.) Theiss.	–	Saprobic	*Spartina alterniflora*	Argentina: Buenos Aires	[36]
**Mytilinidiales**					
**Mytilinidiaceae**					
*Septonema secedens* Corda	Living, senescent, and decaying leaves	Saprobic	*Juncus roemerianus*	USA: Florida	[43]
**Phaeotrichales**					
**Phaeotrichaceae**					
*Trichodelitschia bisporula* (P. Crouan and H. Crouan) E. Müll. and Arx	–	–	*Elymus pungens*	UK	[38]
			*Spartina townsendii*	UK	[38]
**Pleosporales**					
**Amniculicolaceae**					
*Neomassariosphaeria typhicola* (P. Karst.) Y. Zhang ter, J. Fourn. and K.D. Hyde	–	–	*Juncus roemerianus*	–	[35]
	Decaying herbaceous stems	Saprobic	*Spartina densiflora*	Argentina: Buenos Aires	[64]
	–	Saprobic	*Spartina* spp.	Argentina: Buenos Aires	[32,35,36]
	–	Saprobic	Unidentified saltmarsh plants	USA: Mississippi	[58]
**Camarosporiaceae**					
*Camarosporium feurichii* Henn.	Living/decomposing leaf sheaths	Saprobic	*Phragmites australis*	Netherlands: Zeeland	[39]
*Camarosporium palliatum* Kohlm. and E. Kohlm.	–	–	*Sarcocornia perennis*	–	[35]
	–	–	*Salicornia* sp.	–	[35]
	–	–	*Salicornia virginica*	–	[35]
	–	Saprobic or perthophytic	Salt marsh plants	India: Maharashtra	[52]
	–	–	*Suaeda vermiculata*		[35]
*Camarosporium roumeguerei* Sacc.	–	–	*Atripex halimus*		[35]
	–	–	*Atripex* sp.		[35]
	–	–	*Distichlis spicata*		[35]
	Twigs	–	*Salicornia europaea*	France	[35,69]
	–	–	*Sarcocornia fruticosa*		[35]
	–	–	*Salicornia* sp.		[35]
	–	Saprobic or perthophytic	Salt marsh plants	India: Gujarat, Maharashtra, Tamil Nadu, Andhara Pradesh, West Bengal	[52]
	Leaf sheaths and blades, stem	Saprobic	*Spartina maritima*	Portugal: Algarve, Centro	[59]
	–	–	*Suaeda maritima*	–	[35]
*Camarosporium salicorniae* Hansf.	Twigs	–	*Sarcocornia quinqueflora*	South Australia	[62]
*Camarosporium* spp.	Living/decomposing leaf sheaths and stems	Saprobic	*Phragmites australis*	Netherlands: Zeeland	[39,40]
*Camarosporium suaedae-fruticosae* S. Ahmad	Dead branches	Saprobic	*Suaeda vermiculata*	Pakistan	[70]
**Coniothyriaceae**					
*Coniothyrium obiones* Jaap	–	–	*Atriplex portulacoides*	–	[35]
	–	Saprobic	Salt marsh plants	India: Orissa	[52]
	Leaf sheaths and blades, stem	Saprobic	*Spartina maritima*	Portugal: Algarve	[59]
*Coniothyrium* spp.	Living, senescent, and decaying leaves	Saprobic	*Juncus roemerianus*	USA: Florida	[43]
**Cyclothyriellaceae**					
*Massariosphaeria erucacea* Kohlm., Volkm.-Kohlm. and O.E. Erikss.	Senescent culms and leaves	Saprobic	*Juncus roemerianus*	USA: North Carolina	[66]
*Massariosphaeria scirpina* (G. Winter) Leuchtm.	–	Saprobic	*Spartina* sp.	USA: Florida, North Carolina	[71]
*Massariosphaeria* sp.	Living/decomposing stems	Saprobic	*Phragmites australis*	Netherlands: Zeeland	[40]
**Dictyosporiaceae**					
*Dictyosporium oblongum* (Fuckel) S. Hughes	Living/decomposing leaf blades and sheaths, stems	Saprobic	*Phragmites australis*	Netherlands: Zeeland	[39,40,50]
*Dictyosporium pelagicum* (Linder) G.C. Hughes ex E.B.G. Jones	Decomposing culms	Saprobic	*Spartina alterniflora*	USA: Rhode Island	[35,61]
	–	–	*Spartina* spp.	–	[32]
	Leaf sheaths and blades, stem	Saprobic	*Spartina marítima*	Portugal: Alentejo, Lisbon, Algarve, Centro	[54,59,63]
*Jalapriya toruloides* (Corda) M.J. D’souza, Hong Y. Su, Z.L. Luo and K.D. Hyde	Stems	Saprobic	*Spartina* sp.	UK	[72]
**Didymellaceae**					
*Ascochyta* cf. *arundinariae* Tassi	Living/decomposing leaf blades and sheaths	Saprobic	*Phragmites australis*	Netherlands: Zeeland	[39,50]
*Ascochyta leptospora* (Trail) Hara	Living/decomposing leaf sheaths	Saprobic	*Phragmites australis*	Netherlands: Zeeland	[39]
*Ascochyta salicorniae-patulae* (Trotter) Melnik	–	Saprobic, parasitic	*Salicornia* spp.	Canada, Denmark, Germany, India, UK, USA	[52]
*Ascochyta* spp.	Living/decomposing leaf sheaths	Saprobic	*Phragmites australis*	Netherlands: Zeeland	[39]
	Sheath	Saprobic	*Spartina marítima*	Portugal: Alentejo	[54]
*Chaetasbolisia* sp.	Decaying stems and leaf sheaths	Saprobic	*Phragmites australis*	China: Hong Kong	[41]
*Didymella glacialis* Rehm	Living/decomposing leaf blades and sheaths, stems	Saprobic	*Phragmites australis*	Netherlands: Zeeland	[39,40,50]
*Didymella glomerata* (Corda) Qian Chen and L. Cai	Rhizome and basal area	Saprobic	*Spartina densiflora*	Argentina: Buenos Aires	[64]
*Didymella* spp.	Living/decomposing leaf blades and sheaths	Saprobic	*Phragmites australis*	Netherlands: Zeeland	[39,50]
	–	Pathogenic	*Spartina cynosuroides*	USA: Louisiana	[44]
*Epicoccum nigrum* Link	Leaves	Saprobic	*Distichlis spicata*	Argentina: Buenos Aires	[47]
	Living, senescent, and decaying leaves	Saprobic	*Juncus roemerianus*	USA: Florida	[43]
	Inflorescence, upper leaves, seeds	Saprobic, parasitic	*Spartina alterniflora*	USA: Rhode Island, Connecticut, Virginia, Florida, North Carolina	[36,61,73,74]
*Epicoccum* sp.	–	–	*Spartina alterniflora*	–	[35]
*Microsphaeropsis* spp.	Living/decomposing leaf blades and sheaths	Saprobic	*Phragmites australis*	Netherlands: Zeeland	[39,41,50]
*Phoma herbarum* Westend.	Leaves	Saprobic	*Distichlis spicata*	Argentina: Buenos Aires	[47]
*Phoma leveillei* Boerema and G.J. Bollen	Leaves	Saprobic	*Distichlis spicata*	Argentina: Buenos Aires	[47]
*Phoma suaedae* Jaap	Twigs, leaves, stems	Saprobic	*Suaeda maritima*, *Suaeda* sp.	Germany; India	[75]
	–	–	*Suaeda maritima*	–	[35]
*Phoma* spp.	–	–	*Crithmum maritimum*	–	[35]
	–	–	*Atriplex portulacoides*	–	[35]
	Living, senescent, and decaying leaves	Saprobic	*Juncus roemerianus*	USA: Florida	[43]
	Living/decomposing leaf blades and sheaths, stems	Saprobic	*Phragmites australis*	China: Hong Kong; Netherlands: Zeeland	[39,40,41,50]
	–	–	*Salicornia europaea*	–	[35]
	–	–	*Spartina alterniflora*	USA: North Carolina, Rhode Island	[20,35,36,61,73,74]
	–	Saprobic	*Spartina patens*	USA: Rhode Island	[36]
	–	Saprobic	*Spartina* sp.	Argentina: Buenos Aires; Canada; USA: Maine, South Carolina	[36,71]
	–	–	*Spartina townsendii*	UK: England	[35,49,65]
	Leaf sheaths and blades, stem	Saprobic	*Spartina marítima*	Portugal: Alentejo, Lisbon, Algarve, Centro	[54,59,63]
*Paraboeremia putaminum* (Speg.) Qian Chen and L. Cai	Leaves	Saprobic	*Distichlis spicata*	Argentina: Buenos Aires	[47]
*Stagonosporopsis salicorniae* (Magnus) Died.	–	–	*Salicornia europaea*	–	[35]
	–	–	*Salicornia patula*	–	[35]
**Didymosphaeriaceae**					
*Didymosphaeria lignomaris* Strongman and J.D. Mill.	Basal area of the sheath	Saprobic	*Spartina densiflora*	Argentina: Buenos Aires	[64]
	–	–	*Spartina* spp.	–	[32]
*Julella herbatilis* Kohlm., Volkm.-Kohlm. and O.E. Erikss.	Senescent leaves	Saprobic	*Juncus roemerianus*	USA: North Carolina	[76]
*Paraphaeosphaeria apicicola* Kohlm., Volkm.-Kohlm. and O.E. Erikss.	Senescent leaves	Saprobic	*Juncus roemerianus*	USA: North Carolina	[51]
*Paraphaeosphaeria pilleata* Kohlm., Volkm.-Kohlm. and O.E. Erikss.	Senescent culms	Saprobic	*Juncus roemerianus*	USA: North Carolina	[77]
*Paraphaeosphaeria michotii* (Westend.) O.E. Erikss.	–	–	*Elymus pungens*	UK	[38]
	Living/decomposing leaf sheaths	Saprobic	*Phragmites australis*	Netherlands: Zeeland	[39]
*Pseudopithomyces atro-olivaceus* (Cooke and Harkn.) G. Guevara, K.C. Cunha and Gené	Senescent and decaying leaves	Saprobic	*Juncus roemerianus*	USA: Florida	[43]
*Pseudopithomyces chartarum* (Berk. and M.A. Curtis) Jun F. Li, Ariyaw. and K.D. Hyde	Senescent leaves	Saprobic	*Juncus roemerianus*	USA: Florida	[43]
*Pseudopithomyces maydicus* (Sacc.) Jun F. Li, Ariyaw. and K.D. Hyde	Senescent and decaying leaves	Saprobic	*Juncus roemerianus*	USA: Florida	[43]
	Decaying stems and leaf sheaths	Saprobic	*Phragmites australis*	China: Hong Kong	[41]
*Spegazzinia tessarthra* (Berk. and M.A. Curtis) Sacc.	Living leaves		*Juncus roemerianus*	USA: Florida	[43]
	Decaying stems and leaf sheaths	Saprobic	*Phragmites australis*	China: Hong Kong	[41]
*Tremateia halophila* Kohlm., Volkm.-Kohlm. and O.E. Erikss.	Lower and middle parts of senescent culms	Saprobic	*Juncus roemerianus*	USA: North Carolina	[78]
	–	Saprobic	*Spartina marítima*	Portugal: Alentejo, Lisbon	[54]
**Lentitheciaceae**					
*Halobyssothecium estuariae* B. Devadatha, Calabon, K.D. Hyde and E.B.G. Jones	Dead culm	Saprobic	*Phragmites australis*	UK: Pembrokeshire	[79]
*Halobyssothecium obiones* (P. Crouan and H. Crouan) Dayarathne, E.B.G. Jones and K.D. Hyde	Drift stems, attached and dead culms	Saprobic	*Spartina alterniflora*	India: Maharashtra, Tamil Nadu, Andhara Pradesh; USA: Maine, Rhode Island, Connecticut, Massachusetts, New Jersey, Maryland, Virginia, North Carolina, South Carolina, Florida, Mississippi, Texas	[20,35,52,61,71,74,80,81,82]
	–	–	*Spartina cynosuroides*	–	[35]
	Pod and rhizome	Saprobic	*Spartina densiflora*	Argentina: Buenos Aires	[64]
	–	Saprobic	*Spartina patens*	USA: Rhode Island	[36]
	Culms	Saprobic	*Spartina* sp.	UK: England, Hampshire	[79,83]
	Stem	Saprobic	*Spartina townsendii*	UK: Hampshire, Wales	[49,65]
	–	Saprobic	*Spartina* spp.	USA: New Jersey, South Carolina; Mississippi, Argentina: Buenos Aires	[32,35,36,58,84]
	Stem, leaf sheaths, and blades	Saprobic	*Spartina marítima*	Portugal: Alentejo, Lisbon, Algarve, Centro	[31,54,59,63]
	–	Saprobic	Unidentified saltmarsh plants	USA: Mississippi	[55,58]
	–	–	*Elymus pungens*	–	[35]
	–	–	*Atriplex portulacoides*	–	[35]
	–	–	*Spartina townsendii*	–	[35]
*Halobyssothecium phragmitis* M.S. Calabon, E.B.G. Jones, S. Tibell and K.D. Hyde	Dead culm and stem	Saprobic	*Phragmites* sp.	Sweden: Gotland	[85]
*Halobyssothecium versicolor* M.S. Calabon, E.B.G. Jones and K.D. Hyde	Dead stem	Saprobic	*Atriplex portulacoides*	UK: Hampshire	[85]
*Keissleriella culmifida* (P. Karst.) S.K. Bose	–	–	*Elymus pungens*	UK	[38]
*Keissleriella linearis* E. Müll. ex Dennis	Living/decomposing stems	Saprobic	*Phragmites australis*	Netherlands: Zeeland	[40]
	Dead culm	Saprobic	*Phragmites* sp.	Sweden: Gotland	[85]
*Keissleriella phragmiticola* Wanas., E.B.G. Jones and K.D. Hyde	Culms	Saprobic	*Phragmites australis*	UK: Wales	[79]
*Keissleriella rara* Kohlm., Volkm.-Kohlm. and O.E. Erikss.	Senescent culms	Saprobic	*Juncus roemerianus*	USA: North Carolina	[77]
*Keissleriella* spp.	Senescent leaves	Saprobic	*Juncus roemerianus*	USA: Florida	[43]
*Lentithecium fluviatile* (Aptroot and Van Ryck.) K.D. Hyde, J. Fourn. and Ying Zhang	Dead leaf sheaths	Saprobic	*Phragmites australis*	Belgium: East Flanders	[86]
	Living/decomposing leaf blades and sheaths, stems	Saprobic	*Phragmites australis*	Netherlands: Zeeland	[39,40,50]
*Setoseptoria arundinacea* (Sowerby) Kaz. Tanaka and K. Hiray.	–	–	*Elymus pungens*	UK	[38]
	Living/decomposing leaf blades and sheaths, stems	Saprobic	*Phragmites australis*	Netherlands: Zeeland	[39,40,50]
	–	Saprobic	*Spartina* sp.	USA: North Carolina, Florida	[71]
*Setoseptoria phragmitis* Quaedvl., Verkley and Crous	Culm	Saprobic	*Phragmites* sp.	Sweden: Södermanland	[87]
*Towyspora aestuari* Wanas., E.B.G. Jones and K.D. Hyde	–	–	*Phragmites australis*	UK: Wales	[88]
**Leptosphaeriaceae**					
*Leptosphaeria albopunctata* (Westend.) Sacc.	–	–	*Juncus maritimus*	–	[35]
	–	–	*Phragmites australis*	–	[35]
	Attached culms	-	*Spartina alterniflora*	USA: Rhode Island	[35,36,61,71,73,80]
	–	–	*Spartina* spp.	Canada: Bay of Fundy; USA: New Jersey, South Carolina; Argentina: Buenos Aires	[35,36,48,89,90]
	Stem	Saprobic	*Spartina townsendii*	UK: Wales	[35,65]
*Leptosphaeria australiensis* (Cribb and J.W. Cribb) G.C. Hughes	Senescent and decaying leaves	Saprobic	*Juncus roemerianus*	USA: Florida	[43]
	Pod	Saprobic	*Spartina densiflora*	Argentina: Buenos Aires	[64]
	–	–	*Spartina* spp.	–	[32]
*Leptosphaeria culmifraga* (Fr.) Ces. and De Not.	–	–	*Elymus pungens*	UK	[38]
*Leptosphaeria littoralis* Sacc.	–	–	*Elymus pungens*	UK	[38]
*Leptosphaeria marina* Ellis and Everh.	–	–	*Juncus roemerianus*		[35]
	–	Saprobic	*Spartina alterniflora*	USA: Maine, Rhode Island, Connecticut, New Jersey, Delaware, Virginia, North Carolina, South Carolina	[35,36,71,73,80]
	–	Saprobic	*Spartina* spp.	Canada; USA: New Jersey	[32,35,36,65,89,90,91]
	–	–	*Spartina townsendii*	UK	[35,38]
	Leaf sheaths and blades, stem	Saprobic	*Spartina maritima*	Portugal: Algarve	[31,59]
*Leptosphaeria orae-maris* Linder	–	–	*Arundo donax*	–	[35]
	–	Saprobic	*Lysimachia maritima*	USA: Massachusetts	[35,92]
	–	Saprobic	*Spartina alterniflora*	USA: Massachusetts, Rhode Island, North Carolina, Florida, Texas	[36,71,80,92]
	Rhizome	Saprobic	*Spartina densiflora*	Argentina: Buenos Aires	[64]
	–	–	*Spartina* spp.	–	[32]
	–	Saprobic	*Spartina townsendii*	UK	[35,65,93]
*Leptosphaeria pelagica* E.B.G. Jones	–	–	*Elymus pungens*	UK	[35,38]
	–	–	*Puccinellia maritima*	UK	[38]
	Decaying herbaceous stems, dead culms, decaying leaves	Saprobic	*Spartina alterniflora*	USA: Connecticut, Mississippi, Rhode Island; India: Goa, Karanataka	[20,36,52,55,73,94]
	–	Saprobic	*Spartina densiflora*	Argentina: Buenos Aires	[64]
	–	Saprobic	*Spartina patens*	USA: Rhode Island	[36]
	–	–	*Spartina townsendii*	UK	[38]
	–	–	*Spartina* spp.	UK	[32,65]
	Sheath	Saprobic	*Spartina marítima*	Portugal: Alentejo, Lisbon	[54]
	Stem	Saprobic	*Spartina marítima*	Portugal: Alentejo	[63]
*Leptosphaeria peruvianae* Speg.	Decaying stems	Saprobic	*Sarcocornia perennis*	Argentina: Buenos Aires; in temperate marine waters	[52]
*Leptosphaeria* spp.	Decaying leaves	Saprobic	*Juncus roemerianus*	USA: Mississippi	[55]
	Decaying stems and leaf sheaths	Saprobic	*Phragmites australis*	China: Hong Kong	[41]
	–	–	*Spartina alterniflora*	USA: Rhode Island	[74]
	Leaf sheaths and blades, stem	Saprobic	*Spartina maritima*	Portugal: Centro	[59]
*Leptosphaeria suaedae* Hansf.	Dead twigs	Saprobic	*Suaeda australis*	South Australia	[95]
**Lindgomycetaceae**					
*Arundellina typhae* Wanas., E.B.G. Jones and K.D. Hyde	Dead stem	Saprobic	*Typha* sp.	UK: England	[96]
**Lophiostomataceae**					
*Lophiostoma semiliberum* (Desm.) Ces. and De Not.	Living/decomposing stems	Saprobic	*Phragmites australis*	Netherlands: Zeeland	[40]
*Lophiostoma* sp.	–	–	*Elymus pungens*	UK	[38]
*Sigarispora arundinis* (Pers.) Thambug., Qing Tian, Kaz. Tanaka and K.D. Hyde	Living/decomposing stems	Saprobic	*Phragmites australis*	Netherlands: Zeeland	[40]
**Massarinaceae**					
*Helminthosporium* sp.	Decaying leaf blades	Saprobic	*Spartina alterniflora*	USA: Georgia	[56]
*Massarina carolinensis* Kohlm., Volkm.-Kohlm. and O.E. Erikss.	Senescent culms	Saprobic	*Juncus roemerianus*	USA: North Carolina	[77]
*Massarina igniaria* (C. Booth) Aptroot	Decaying leaves	Saprobic	*Juncus roemerianus*	USA: Florida	[43]
*Massarina phragmiticola* Poon and K.D. Hyde	Decaying stems and leaf sheaths	Saprobic	*Phragmites australis*	China: Hong Kong	[41]
*Massarina ricifera* Kohlm., Volkm.-Kohlm. and O.E. Erikss.	Lower parts of senescent culms, decaying leaves	Saprobic	*Juncus roemerianus*	USA: Alabama, Mississippi, North Carolina	[55,58,97]
*Massarina* spp.	Senescent and decaying leaves	Saprobic	*Juncus roemerianus*	USA: Florida	[43]
	Living/decomposing leaf sheaths	Saprobic	*Phragmites australis*	Netherlands: Zeeland	[39]
*Stagonospora abundata* Kohlm. and Volkm.-Kohlm.	Senescent leaves and bracts	Saprobic	*Juncus roemerianus*	USA: Florida, Georgia, North Carolina	[98]
*Stagonospora cylindrica* Gunnell	Living/decomposing stems	Saprobic	*Phragmites australis*	Netherlands: Zeeland	[40]
*Stagonospora elegans* (Berk.) Sacc. and Traverso	Living/decomposing leaf sheaths, stems, culms	Saprobic	*Phragmites australis*	Australis; Netherlands: Zeeland	[39,40,95]
*Stagonospora epicalamia* (Cooke) Sacc.	–	–	*Phragmites australis*	Australia	[95]
*Stagonospora haliclysta* Kohlm.	Leaf sheaths and blades, stem	Saprobic	*Spartina maritima*	Portugal: Algarve	[59]
*Stagonospora* spp.	Living and senescent leaves	Saprobic	*Juncus roemerianus*	USA: Florida	[43]
	Living/decomposing leaf blades and sheaths, stems	Saprobic	*Phragmites australis*	China: Hong Kong; Netherlands: Zeeland	[39,40,41,50]
	Senescent and dead leaves/inflorescence, living and dead seeds, decaying leaf blades	Saprobic, pathogenic	*Spartina alterniflora*	Canada; USA: Maine, Rhode Island, Georgia, Connecticut, New Jersey, Virginia, Florida, North Carolina; Argentina: Buenos Aires	[35,36,56,73,74]
	–	Pathogenic	*Spartina cynosuroides*	USA: Maryland	[44]
	–	Saprobic	*Spartina patens*	USA: Rhode Island	[35,36]
	–	Saprobic	*Spartina* spp.	Canada	[35,36]
	Leaf sheaths and blades, stem, limb	Saprobic	*Spartina marítima*	Portugal: Alentejo, Lisbon, Algarve, Centro	[31,54,59]
*Stagonospora suaedae* Syd. and P. Syd.	Leaves	–	*Suaeda marítima*	Germany	[99]
**Melanommataceae**					
*Aposphaeria* spp.	Living/decomposing leaf sheaths, stems	Saprobic	*Phragmites australis*	Netherlands: Zeeland	[39,40]
*Bicrouania maritima* (P. Crouan and H. Crouan) Kohlm. and Volkm.-Kohlm.	Dead stems	Saprobic	*Atriplex portulacoides*	India	[35,52]
**Morosphaeriaceae**					
*Helicascus kanaloanus* Kohlm.	–	–	*Spartina* spp.	–	[32]
**Neocamarosporiaceae**					
*Neocamarosporium artemisiae* Dayarathne and E.B.G. Jones	–	Saprobic	*Artemisia maritima*	Sweden: Bohuslän	[100]
*Neocamarosporium maritimae* Dayarathne and E.B.G. Jones	–	Saprobic	*Artemisia maritima*	Sweden: Bohuslän	[100]
*Neocamarosporium obiones* (Jaap) Wanas. and K.D. Hyde	–	–	*Atriplex portulacoides*	–	[35]
*Neocamarosporium phragmitis* D.N. Wanasinghe, E.B.G. Jones and K.D. Hyde	Decaying culms	Saprobic	*Phragmites australis*	UK	[101]
*Neocamarosporium salicorniicola* Dayar., E.B.G. Jones and K.D. Hyde	Dead stems	Saprobic	*Salicornia* sp.	Thailand	[102]
**Periconiaceae**					
*Periconia cookei* E.W. Mason and M.B. Ellis	Senescent and decaying leaves	Saprobic	*Juncus roemerianus*	USA: Florida	[43]
	Living/decomposing leaf blades and sheaths	Saprobic	*Phragmites australis*	Netherlands: Zeeland	[39,50]
*Periconia digitata* (Cooke) Sacc.	Living, senescent, and decaying leaves	Saprobic	*Juncus roemerianus*	USA: Florida	[43]
*Periconia digitata* (Cooke) Sacc.	Living/decomposing leaf sheaths	Saprobic	*Phragmites australis*	Netherlands: Zeeland	[39]
*Periconia echinochloae* (Bat.) M.B. Ellis	Senescent and decaying leaves	Saprobic	*Juncus roemerianus*	USA: Florida	[43]
*Periconia minutissima* Corda	Leaves	Saprobic	*Distichlis spicata*	Argentina: Buenos Aires	[47]
	Senescent and decaying leaves	Saprobic	*Juncus roemerianus*	USA: Florida	[43]
	Living/decomposing leaf sheaths	Saprobic	*Phragmites australis*	Netherlands: Zeeland	[39]
*Periconia* sp.	–	Saprobic	Unidentifed saltmarsh plants	USA: Mississippi	[58]
**Phaeosphaeriaceae**					
*Amarenomyces ammophilae* (Lasch) O.E. Erikss.	–	–	*Ammophila arenaria*	–	[35]
	–	–	*× Ammocalamagrostis baltica*	–	[35]
	–	–	*Uniola paniculata*	–	[35]
*Amphisphaeria culmicola* Sacc.	Stem		*Spartina townsendii*	UK: England	[49]
*Camarosporioides phragmitis* W.J. Li and K.D. Hyde	Dead stem	Saprobic	*Phragmites australis*	Germany	[96]
*Hendersonia culmiseda* Sacc.	Living/decomposing leaf blades	Saprobic	*Phragmites australis*	Netherlands: Zeeland	[50]
	Living/decomposing leaf sheaths	Saprobic	*Phragmites australis*	Netherlands: Zeeland	[39]
	–	–	*Spartina townsendii*	UK	[103]
*Hendersonia* spp.	Living/decomposing leaf blades and sheaths	Saprobic	*Phragmites australis*	Netherlands: Zeeland; USA: Florida	[39,43,50]
*Loratospora aestuarii* Kohlm. and Volkm.-Kohlm.	Senescent culms	Saprobic	*Juncus roemerianus*	USA: North Carolina	[104]
*Loratospora aestuarii* Kohlm. and Volkm.-Kohlm.	–	Saprobic	Unidentified saltmarsh plants	USA: Mississippi	[58]
*Ophiobolus littoralis* (P. Crouan and H. Crouan) Sacc.	–	–	*Elymus pungens*	UK	[38]
*Phaeoseptoria* sp.	Living/decomposing leaf sheaths	Saprobic	*Phragmites australis*	Netherlands: Zeeland	[39]
*Phaeosphaeria anchiala* Kohlm., Volkm.-Kohlm. and C.K.M. Tsui	Senescent leaves	Saprobic	*Juncus roemerianus*	USA: Florida, Georgia, Maryland, North Carolina, Virginia	[105]
*Phaeosphaeria caricinella* (P. Karst.) O.E. Erikss.	–	–	*Spartina* sp.	USA: Florida, North Carolina	[71]
*Phaeosphaeria culmorum* (Auersw.) Leuchtm.	Living/decomposing leaf blades and sheaths	Saprobic	*Phragmites australis*	Netherlands: Zeeland	[39,50]
*Phaeosphaeria eustoma* (Fuckel) L. Holm	Living/decomposing leaf blades and sheaths, stems, culms	Saprobic	*Phragmites australis*	Netherlands: Zeeland	[39,40,50,95]
*Phaeosphaeria fuckelii* (Niessl) L. Holm	–	–	*Elymus pungens*	UK	[38]
*Phaeosphaeria gessneri* Shoemaker and C.E. Babc.	–	–	*Spartina* spp.	–	[32]
*Phaeosphaeria halima* (T.W. Johnson) Shoemaker and C.E. Babc.	Dead culms; Decaying leaves, leaf blades	Saprobic	*Spartina alterniflora*	India: Kerala; USA: California, Georgia, Mississippi, Vancouver, North Carolina	[25,35,52,55,56,57,58,71,80]
	Decaying leaf blades	Saprobic	*Spartina densiflora*	USA: California	[25]
			*Spartina* spp.		[32]
	Decaying leaves	Saprobic	*Spartina foliosa*	USA: California	[25]
	Leaf sheaths and blades, stem	Saprobic	*Spartina maritima*	Portugal: Algarve, Centro	[31]
*Phaeosphaeria herpotrichoides* (De Not.) L. Holm	–	–	*Spartina patens*	USA: North Carolina, Florida	[71]
*Phaeosphaeria juncina* (Auersw.) L. Holm	–	Saprobic	*Juncus roemerianus*	USA: Florida	[43]
*Phaeosphaeria luctuosa* (Niessl ex Sacc.) Y. Otani and Mikawa	Living/decomposing leaf sheaths, stems	Saprobic	*Phragmites australis*	Netherlands: Zeeland	[39,40]
	–	–	*Elymus pungens*	UK	[38]
*Phaeosphaeria macrosporidium* (E.B.G. Jones) Shoemaker and C.E. Babc.	Decaying stems	Saprobic	*Spartina sp*	UK: Wales, England	[65]
	Stem	Saprobic	*Spartina marítima*	Portugal: Lisbon	[54,63]
*Phaeosphaeria microscopica* (P. Karst.) O.E. Erikss.	–	–	*Elymus pungens*	UK	[38]
*Phaeosphaeria neomaritima* (R.V. Gessner and Kohlm.) Shoemaker and C.E. Babc.	–	–	*Juncus maritimus*	–	[35]
	–	–	*Juncus roemerianus*	–	[35]
	–	Saprobic	*Juncus* sp.	Canada; India: Maharashtra, Karnataka; USA: Virginia, North Carolina	[36,52,71,80]
	–	–	*Spartina alterniflora*	–	[35]
	–	Saprobic	*Spartina* spp.	Canada; USA: North Carolina, Virginia	[32,71,80]
	–	–	*Spartina townsendii*	UK	[35,93]
	Stem	Saprobic	*Spartina marítima*	Portugal: Alentejo	[63]
*Phaeosphaeria nigrans* (Roberge ex Desm.) L. Holm	–	–	*Elymus pungens*	UK	[38]
*Phaeosphaeria olivacea* Kohlm., Volkm.-Kohlm. and O.E. Erikss.	Senescent leaves	Saprobic	*Juncus roemerianus*	USA: North Carolina, Mississippi	[58,76]
*Phaeosphaeria pontiformis* (Fuckel) Leuchtm.	–	–	*Elymus pungens*	UK	[38]
	Living/decomposing leaf blades and sheaths, stems	Saprobic	*Phragmites australis*	Netherlands: Zeeland	[39,40,50]
*Phaeosphaeria roemeriani* Kohlm., Volkm.-Kohlm. and O.E. Erikss.	Senescent and decaying leaves	Saprobic	*Juncus roemerianus*	USA: Mississippi, North Carolina	[55,58,60]
*Phaeosphaeria spartinae* (Ellis and Everh.) Shoemaker and C.E. Babc.	–	Saprobic	*Spartina* spp.	India: Kerala	[32,52]
	Decaying herbaceous stems and pod	Saprobic	*Spartina densiflora*	Argentina: Buenos Aires	[64]
	–	Saprobic	*Spartina marítima*	Portugal: Lisbon	[54]
*Phaeosphaeria spartinicola* Leuchtm.	–	Saprobic	*Juncus* sp.	India	[52]
	Dead leaves, decaying leaf blades	Saprobic	*Spartina alterniflora*	Mexico; USA: Alabama, California, Georgia, Mississippi; Canada: Nova Scotia, New Brunswick	[25,36,55,56,57,58]
	Pod, leaf blades	Saprobic	*Spartina densiflora*	Argentina: Buenos Aires; USA: California	[25,64]
	–	–	*Spartina* spp.	–	[32]
	Leaf blades	Saprobic	*Spartina foliosa*	USA: California	[25]
	Leaf sheaths and blades, stem, limb	Saprobic	*Spartina marítima*	Portugal: Alentejo, Lisbon, Algarve, Centro	[31,54,59,63]
*Phaeosphaeria* spp.	Living/decomposing leaf blades and sheaths, stems	Saprobic	*Phragmites australis*	Netherlands: Zeeland	[39,40,50]
	–	Saprobic	*Spartina alterniflora*	USA: Rhode Island	[74]
*Sclerostagonospora* sp.	Decaying stems and leaf sheaths	Saprobic	*Phragmites australis*	China: Hong Kong	[41]
*Septoriella phragmitis* Oudem.	Living/decomposing leaf sheaths and stems	Saprobic	*Phragmites australis*	Netherlands: Zeeland	[39,40]
*Septoriella* spp.	Decaying stems and leaf sheaths and blades, stems	Saprobic	*Phragmites australis*	China: Hong Kong; Netherlands: Zeeland	[39,40,41,50]
*Septoriella thalassica* (Speg.) Nag Raj	–	–	*Distichlis spicata*	–	[35]
			*Distichlis spicata*		[35]
*Septoriella unigalerita* Kohlm. and Volkm.-Kohlm.	Senescent leaves	Saprobic	*Juncus roemerianus*	USA: North Carolina	[98]
*Septoriella vagans* (Niessl) Y. Marín and Crous	–	–	*Elymus pungens*	UK	[38]
	–	–	*Puccinellia maritima*	UK	[38]
	–	Saprobic	*Spartina alterniflora*	USA: Rhode Island	[74]
**Pleomassariaceae**					
*Splanchnonema* sp.	Living, senescent, and decaying leaves	Saprobic	*Juncus roemerianus*	USA: Florida	[43]
**Pleosporaceae**					
*Alternaria alternata* (Fr.) Keissl.	Leaves	Saprobic	*Distichlis spicata*	Argentina: Buenos Aires	[47]
	Living, senescent, and decaying leaves	Saprobic	*Juncus roemerianus*	USA: Florida	[43]
	Living/decomposing leaf blades and sheaths, stems	Saprobic	*Phragmites australis*	Netherlands: Zeeland	[39,41,50]
	–	Saprobic	*Spartina alterniflora*	USA: North Carolina	[74]
	Leaves, stems, and roots	Saprobic	*Spartina* sp.	Canada: Bay of Fundy	[48]
*Alternaria infectoria* E.G. Simmons	–	–	*Elymus pungens*	UK	[38]
	Living/decomposing leaf sheaths	Saprobic	*Phragmites australis*	Netherlands: Zeeland	[39]
*Alternaria longissima* Deighton and MacGarvie	Living, senescent, and decaying leaves	Saprobic	*Juncus roemerianus*	USA: Florida	[43]
*Alternaria maritima* G.K. Sutherl.	Stem	Saprobic, pathogenic	*Spartina townsendii*	UK: England	[49]
*Alternaria* spp.	–	–	*Atriplex portulacoides*	–	[35]
	–	–	*Juncus roemerianus*	–	[35]
	–	–	*Salsola kali*	–	[35]
	Inflorescence and upper leaves	Saprobic, parasitic	*Spartina alterniflora*	USA: Rhode Island	[35,61]
	Culms	Saprobic	*Spartina* sp.	Thailand	This study
	–	–	*Spartina townsendii*	–	[35]
*Bipolaris cynodontis (Marignoni) Shoemaker*	Leaves	Saprobic	*Distichlis spicata*	Argentina: Buenos Aires	[47]
*Curvularia hawaiiensis* (Bugnic. ex M.B. Ellis) Manamgoda, L. Cai and K.D. Hyde	Living and senescent leaves	Saprobic	*Juncus roemerianus*	USA: Florida	[43]
	Decaying stems and leaf sheaths	Saprobic	*Phragmites australis*	China: Hong Kong	[41]
*Curvularia protuberata* R.R. Nelson and Hodges	Leaves	Saprobic	*Distichlis spicata*	Argentina: Buenos Aires	[47]
	Senescent leaves	Saprobic	*Juncus roemerianus*	USA: Florida	[43]
*Curvularia* spp.	Living, senescent, and decaying leaves	Saprobic	*Juncus roemerianus*	USA: Florida	[43]
	–	Saprobic	*Spartina altrerniflora*	USA: North Carolina	[74]
*Curvularia tuberculata* B.L. Jain	Senescent and decaying leaves	Saprobic	*Juncus roemerianus*	USA: Florida	[43]
*Decorospora gaudefroyi* (Pat.) Inderb., Kohlm. and Volkm.-Kohlm.	Stems	Saprobic	*Atriplex* sp.	UK: Portsmouth	[106]
	–	–	*Atriplex portulacoides*	–	[35]
	–	–	*Sarcocornia perennis*	–	[35]
	–	–	*Sarcoconia fructicosa*	–	[35]
	–	–	*Salicornia europaea*	–	[35]
	–	–	*Salicornia* sp.	–	[35]
	Leaf sheaths and blades, stem	Saprobic	*Spartina maritima*	Portugal: Algarve	[59]
	–	–	*Suaeda maritima*	–	[35]
*Drechslera* sp.	Living, senescent, and decaying leaves	Saprobic	*Juncus roemerianus*	USA: Florida	[43]
*Exserohilum rostratum* (Drechsler) K.J. Leonard and Suggs	–	–	*Distichlis spicata*	–	[35]
	Living, senescent, and decaying leaves	Saprobic	*Juncus roemerianus*	USA: Florida	[43]
	Senescent and dead leaves	Saprobic	*Spartina alterniflora*	USA: Rhode Island, North Carolina, Florida	[35,36,73]
	–	–	*Spartina* spp.	–	[32]
*Paradendryphiella arenariae* (Nicot) Woudenb. and Crous	Decomposing culms	Saprobic	*Spartina alterniflora*	USA: Rhode Island	[35,61]
	–	–	*Spartina* spp.	–	[32]
*Paradendryphiella salina* (G.K. Sutherl.) Woudenb. and Crous	–	–	*Atriplex portulacoides*	–	[35]
	Decaying leaves	Saprobic	*Juncus roemerianus*	USA: Florida	[43]
	–	–	*Puccinellia maritima*	–	[35]
	–	–	*Salicornia europaea*	–	[35]
	Decomposing culms	Saprobic	*Spartina alterniflora*	USA: Rhode Island	[35,61]
	–	–	*Spartina* spp.	–	[32]
	–	–	*Spartina townsendii*	–	[35]
	Leaves and stems	Saprobic	*Spartina* sp.	Canada: Bay of Fundy	[48]
	–	–	*Suaeda maritima*	–	[35]
*Pleospora abscondita* Sacc. and Roum.	Living/decomposing leaf sheaths	Saprobic	*Phragmites australis*	Netherlands: Zeeland	[39]
*Pleospora pelagica* T.W. Johnson	Decomposing culms; decaying leaf blades	Saprobic	*Spartina alterniflora*	India: Maharashtra, Kerala; USA: Georgia, Rhode Island, North Carolina, Florida	[35,36,52,56,71,73,74,80]
	Decaying leaf blades	Saprobic	*Spartina densiflora*	USA: California	[25]
		Saprobic	*Spartina* spp.	USA: South Carolina	[32,36]
			*Typha* sp.		[35]
*Pleospora pelvetiae* G.K. Sutherl.	–	Saprobic	Unidentifed saltmarsh plants	USA: Mississippi	[58]
*Pleospora* spp.	–	–	*Salicornia virginica*	–	[35]
	Dead leaves/culms	Saprobic	*Spartina alterniflora*	USA: Rhode Island	[61]
*Pleospora spartinae* (J. Webster and M.T. Lucas) Apinis and Chesters	Decaying stems and leaf sheaths	Saprobic	*Phragmites australis*	China: Hong Kong	[41]
	Decaying leaf blades	Saprobic	*Spartina alterniflora*	USA: Georgia	[56]
	Stem	Saprobic	*Spartina* spp.	Canada: Bay of Fundy	[32,48]
	–	–	*Spartina townsendii*	UK	[35,38,107]
*Pleospora straminis* Sacc. and Speg.	–	–	*Elymus pungens*	UK	[38]
*Pleospora vagans* Niessl var. vagans	Living/decomposing leaf sheaths	Saprobic	*Phragmites australis*	Netherlands: Zeeland	[39]
	Dead culms	Saprobic	*Spartina alterniflora*	USA: Rhode Island	[73]
*Pyrenophora tritici-repentis* (Died.) Drechsler	–	–	*Elymus pungens*	UK	[38]
*Stemphylium botryosum* Wallr.	Leaves	Saprobic	*Distichlis spicata*	Argentina: Buenos Aires	[47]
*Stemphylium lycopersici* (Enjoji) W. Yamam.	Living leaves	–	*Juncus roemerianus*	USA: Florida	[43]
*Stemphylium maritimum* T.W. Johnson	–	Saprobic	*Spartina* sp.	UK	[65]
*Stemphylium* spp.	–	–	*Salsola kali*	–	[35]
	Leaves	Saprobic	*Spartina* spp.	Canada: Bay of Fundy	[35,48]
*Stemphylium vesicarium* (Wallr.) E.G. Simmons	–	–	*Elymus pungens*	UK	[38]
	Living, senescent and decaying leaves	Saprobic	*Juncus roemerianus*	USA: Florida	[43]
	–	Saprobic	*Lysimachia maritima*	USA: Massachusetts	[92]
	–	Saprobic	*Spartina alterniflora*	USA: Rhode Island	[61]
	Glumes, rachis	–	*Spartina townsendii*	UK: England	[38,49]
			*Spartina* sp.	UK	[65]
*Stemphylium triglochinicola* B. Sutton and Piroz.	–	–	*Triglochin maritima*	Sweden: Västergötland	[35,87]
	Dead leaves and inflorescences	Saprobic	*Triglochin* sp.	India: Kerala; UK	[52,108]
*Typhicola typharum* (Desm.) Crous	Senescent and dead leaves	Saprobic, pathogenic	*Spartina alterniflora*	Canada; USA: Maine, Rhode Island, Connecticut, New Jersey, Virginia, North Carolina, Florida	[35,36,61,73,74]
	–	Saprobic	*Spartina patens*	USA: Rhode Island	[36]
	–		*Spartina townsendii*	UK	[38]
	–	Saprobic	*Spartina* spp.	Argentina: Buenos Aires; Canada; USA: Maine	[35,36]
	Stems	Saprobic	*Spartina townsendii*	UK: England	[35,49,65]
**Pleosporales genera *incertae sedis***					
*Phialophorophoma litoralis* Linder	Stem and sheath	Saprobic	*Spartina marítima*	Portugal: Alentejo, Lisbon	[54,63]
*Phialophorophoma* spp.	Living/decomposing leaf sheaths, stems	Saprobic	*Phragmites australis*	Netherlands: Zeeland	[39,40]
*Pyrenochaeta* sp.	Living leaves	Saprobic	*Juncus roemerianus*	USA: Florida	[43]
*Scolecobasidium humicola* G.L. Barron and L.V. Busch	Living, senescent, and decaying leaves	Saprobic	*Juncus roemerianus*	USA: Florida	[43]
**Roussoellaceae**					
*Cytoplea* sp.	Decaying stems and leaf sheaths	Saprobic	*Phragmites australis*	China: Hong Kong	[41]
**Sporormiaceae**					
*Preussia funiculata* (Preuss) Fuckel	–	–	*Spartina townsendii*	UK	[38]
*Preussia terricola* Cain	–	–	*Elymus pungens*	UK	[38]
*Sporormia longipes* Massee and E.S. Salmon	–	–	*Elymus pungens*	UK	[38]
*Sporormia* sp.	Senescent and decaying leaves	Saprobic	*Juncus roemerianus*	USA: Florida	[43]
*Sporormiella intermedia* (Auersw.) S.I. Ahmed and Cain ex Kobayasi	–	–	*Elymus pungens*	UK	[38]
*Sporormiella lageniformis* (Fuckel) S.I. Ahmed and Cain	–	–	*Spartina townsendii*	UK	[38]
*Sporormiella minima* (Auersw.) S.I. Ahmed and Cain	–	–	*Elymus pungens*	UK	[38]
	–	–	*Spartina townsendii*	UK	[38]
**Teichosporaceae**					
*Teichospora striata* (Kohlm. and Volkm.-Kohlm.) Jaklitsch and Voglmayr	Senescent leaves and inflorescences	Saprobic	*Juncus roemerianus*	USA: North Carolina, Virginia	[98]
*Teichospora suaedae* Speg.	Dead branches	Saprobic	*Suaeda divaricata*	Argentina: Mendoza	[109]
Testudinaceae					
*Verruculina enalia* (Kohlm.) Kohlm. and Volkm.-Kohlm.	Decaying stems and leaf sheaths	Saprobic	*Phragmites australis*	China: Hong Kong	[41]
Tetraplosphaeriaceae					
*Tetraploa aristata* Berk. and Broome	Leaves	Saprobic	*Distichlis spicata*	Argentina: Buenos Aires	[47]
	Senescent and decaying leaves	Saprobic	*Juncus roemerianus*	USA: Florida	[43]
	Decaying stems and leaf sheaths	Saprobic	*Phragmites australis*	China: Hong Kong	[41]
**Torulaceae**					
*Torula herbarum* (Pers.) Link	Decaying leaves	Saprobic	*Juncus roemerianus*	USA: Florida	[43]
Trematosphaeriaceae					
*Halomassarina thalassiae* (Kohlm. and Volkm.-Kohlm.) Suetrong, Sakay., E.B.G. Jones, Kohlm., Volkm.-Kohlm. and C.L. Schoch	Decaying stems and leaf sheaths	Saprobic	*Phragmites australis*	China: Hong Kong	[41]
**EUROTIOMYCETES**					
**Chaetothyriales**					
**Herpotrichiellaceae**					
*Rhinocladiella* spp.	Living, senescent, and decaying leaves	Saprobic	*Juncus roemerianus*	USA: Florida	[43]
	Decaying stems and leaf sheaths	Saprobic	*Phragmites australis*	China: Hong Kong	[41]
*Veronaea* sp.	Decaying leaves	Saprobic	*Juncus roemerianus*	USA: Florida	[43]
**Eurotiales**					
**Aspergillaceae**					
*Aspergillus fumigatus* Fresen.	–	–	*Elymus pungens*	UK	[38]
*Aspergillus nidulans* (Eidam) G. Winter	–	–	*Elymus pungens*	UK	[38]
	–	–	*Spartina townsendii*	UK	[38]
*Aspergillus niger* Tiegh.	Living, senescent, and decaying leaves	Saprobic	*Juncus roemerianus*	USA: Florida	[43]
*Aspergillus* spp.	Living, senescent, and decaying leaves	Saprobic	*Juncus roemerianus*	USA: Florida	[43]
	–	–	*Spartina townsendii*	UK: England	[49]
*Monascus purpureus* Went	–	–	*Elymus pungens*	UK	[38]
*Penicillium aurantiogriseum* Dierckx	Leaves	Saprobic	*Spartina* sp.	Canada: Bay of Fundy	[48]
*Penicillium brevicompactum* Dierckx	Roots	Saprobic	*Spartina* sp.	Canada: Bay of Fundy	[48]
*Penicillium chrysogenum* Thom	Roots	Saprobic	*Spartina* sp.	Canada: Bay of Fundy	[48]
*Penicillium lividum* Westling	Leaves and stems	Saprobic	*Spartina* sp.	Canada: Bay of Fundy	[48]
*Penicillium* spp.	Living, senescent, and decaying leaves	Saprobic	*Juncus roemerianus*	USA: Florida	[43]
	Decaying stems and leaf sheaths	Saprobic	*Phragmites australis*	China: Hong Kong	[41]
	–	–	*Spartina townsendii*	UK: England	[49]
**Thermoascaceae**					
*Thermoascus crustaceus* (Apinis and Chesters) Stolk	–	–	*Elymus pungens*	UK	[38]
*Paecilomyces* spp.	Senescent and decaying leaves	Saprobic	*Juncus roemerianus*	USA: Florida	[43]
	Decaying stems and leaf sheaths	Saprobic	*Phragmites australis*	China: Hong Kong	[41]
	–	Saprobic	Salt marsh plants	India: Goa	[52]
**Trichocomaceae**					
*Thermomyces dupontii* (Griffon and Maubl.) Houbraken and Samson	–	–	*Elymus pungens*	UK	[38]
**Onygenales**					
**Onygenaceae**					
*Amauroascus albicans* (Apinis) Arx	–	–	*Elymus pungens*	UK	[38]
*Amauroascus albicans* (Apinis) Arx	–	–	*Spartina townsendii*	UK	[38]
					
**LECANOROMYCETES**					
**Ostropales**					
**Stictidaceae**					
*Glomerobolus gelineus* Kohlm. and Volkm.-Kohlm. *Stictis* sp.	Senescent culms	Saprobic	*Juncus roemerianus*	USA: North Carolina	[110]
	Living/decomposing leaf sheaths	Saprobic	*Phragmites australis*	Netherlands: Zeeland	[39]
					
**LEOTIOMYCETES**					
**Helotiales**					
**Amorphothecaceae**					
*Amorphotheca resinae* Parbery	Roots	Saprobic	*Spartina* sp.	Canada: Bay of Fundy	[48]
**Calloriaceae**					
*Cistella fugiens* (W. Phillips) Matheis	Living/decomposing stems	Saprobic	*Phragmites australis*	Netherlands: Zeeland	[40]
**Helotiaceae**					
*Cyathicula culmicola* (Desm.) De Not.	–	–	*Elymus pungens*	UK	[38]
*Helotium* sp.	–	–	*Elymus pungens*	UK	[38]
**Lachnaceae**					
*Brunnipila palearum* (Desm.) Baral	–	–	*Elymus pungens*	UK	[38]
	–	–	*Spartina townsendii*	UK	[38]
*Lachnum controversum* (Cooke) Rehm	–	–	*Elymus pungens*	UK	[38]
*Lachnum spartinae* S.A. Cantrell	Decaying leaf sheaths	Saprobic	*Spartina alterniflora*	USA: Georgia	[56,111]
	–	–	*Spartina* spp.	–	[32]
**Mollisiaceae**					
*Belonopsis atriella* (Cooke) Lindau	–	–	*Spartina cynosuroides*	USA: Louisiana	[68,90,112]
*Mollisia hydrophila* (P. Karst.) Sacc.	Living/decomposing leaf sheaths	Saprobic	*Phragmites australis*	Netherlands: Zeeland	[39]
*Mollisia palustris* (P. Karst.) P. Karst.	Living/decomposing leaf sheaths	Saprobic	*Phragmites australis*	Netherlands: Zeeland	[39]
*Trichobelonium kneiffii* (Wallr.) J. Schröt.	Living/decomposing leaf sheaths, stems	Saprobic	*Phragmites australis*	Netherlands: Zeeland	[39,40]
**Ploettnerulaceae**					
*Cadophora melinii* Nannf.	Leaves	Saprobic	*Spartina* sp.	Canada: Bay of Fundy	[48]
**Sclerotiniaceae**					
*Botrytis cinerea* Pers.	Stem		*Spartina townsendii*	UK: England	[49]
	Leaves	Saprobic	*Spartina* sp.	Canada: Bay of Fundy	[48]
*Monilia* sp.	Decaying leaves	Saprobic	*Juncus roemerianus*	USA: Florida	[43]
**Solenopeziaceae**					
*Halenospora varia* (Anastasiou) E.B.G. Jones	Senescent leaves	Saprobic	*Juncus roemerianus*	USA: Florida	[43]
	Basal area of the sheath	Saprobic	*Spartina densiflora*	Argentina: Buenos Aires	[64]
	–	–	*Spartina* spp.	–	[32]
**Helotiales genera *incertae sedis***					
*Cejpia hystrix* (De Not.) Baral	–	–	*Elymus pungens*	UK	[38]
*Dactylaria* sp.	Decaying stems and leaf sheaths	Saprobic	*Phragmites australis*	China: Hong Kong	[41]
*Crocicreas gramineum* (Fr.) Fr.	–	–	*Elymus pungens*	UK	[38]
**Leotiales**					
**Leotiales genera *incertae sedis***					
*Flagellospora* sp.	Living leaves	–	*Juncus roemerianus*	USA: Florida	[43]
**Rhytismatales**					
**Rhytismataceae**					
*Lophodermium arundinaceum* (Schrad.) Chevall.	–	–	*Elymus pungens*	UK	[38]
	Living/decomposing leaf sheaths, stems	Saprobic	*Phragmites australis*	Netherlands: Zeeland	[39,40]
**Thelebolales**					
**Thelebolaceae**					
*Thelebolus crustaceus* (Fuckel) Kimbr.	–	–	*Elymus pungens*	UK	[38]
	–	–	*Puccinellia maritima*	UK	[38]
	–	–	*Spartina townsendii*	UK	[38]
					
**ORBILIOMYCETES**					
**Orbiliales**					
**Orbiliaceae**					
*Arthrobotrys conoides* Drechsler	Decaying stems and leaf sheaths	Saprobic	*Phragmites australis*	China: Hong Kong	[41]
*Arthrobotrys* sp.	Decaying stems and leaf sheaths	Saprobic	*Phragmites australis*	China: Hong Kong	[41]
*Orbilia junci* Kohlm., Baral and Volkm.-Kohlm.	Tips of senescent leaves	–	*Juncus roemerianus*	USA: North Carolina	[113]
					
**PEZIZOMYCETES**					
**Pezizales**					
**Pezizaceae**					
*Belonium heteromorphum* (Ellis and Everh.) Seaver	–	–	*Spartina cynosuroides*	USA: Louisiana	[68,114]
					
**SACCHAROMYCETES**					
**Saccharomycetales**					
**Debaryomycetaceae**					
*Debaryomyces hansenii* (Zopf) Lodder and Kreger-van Rij	Decaying leaf blades	Saprobic	*Spartina alterniflora*	USA: Louisiana	[56]
*Scheffersomyces spartinae* (Ahearn, Yarrow and Meyers) Kurtzman and M. Suzuki	Decaying leaf blades	Saprobic	*Spartina alterniflora*	USA: Louisiana	[56]
**Saccharomycetaceae**					
*Kluyveromyces lactis* (Stell.-Dekk.) Van der Walt	Decaying leaf blades	Saprobic	*Spartina alterniflora*	USA: Louisiana	[56]
					
**SORDARIOMYCETES**					
**Amphisphaeriales**					
**Amphisphaeriaceae**					
*Massariella* sp.	–	–	*Spartina townsendii*	UK	[38]
*Ommatomyces coronatus* Kohlm., Volkm.-Kohlm. and O.E. Erikss.	Lower parts of senescent culms	Saprobic	*Juncus roemerianus*	USA: North Carolina	[97]
*Pestalotia* sp.	Living, senescent and decaying leaves	Saprobic	*Juncus roemerianus*	USA: Florida	[43]
**Apiosporaceae**					
*Arthrinium arundinis* (Corda) Dyko and B. Sutton	Living/decomposing leaf sheaths	Saprobic	*Phragmites australis*	Netherlands: Zeeland	[39]
	Dead culms	Saprobic	*Phragmites* sp.	South Australia	[62]
*Arthrinium phaeospermum* (Corda) M.B. Ellis	Living/decomposing leaf blades and sheaths, stems	Saprobic	*Phragmites australis*	Netherlands: Zeeland	[39,40,50]
	–	Saprobic	*Spartina patens*	USA: Rhode Island	[61]
	Inflorescence and upper leaves	Saprobic	*Spartina alterniflora*	USA: Rhode Island	[36]
*Arthrinium* spp.	Living leaves	Saprobic	*Juncus roemerianus*	USA: Florida	[43]
	Decaying stems and leaf sheaths	Saprobic	*Phragmites australis*	China: Hong Kong	[41]
*Nigrospora oryzae* (Berk. and Broome) Petch	Leaves	Saprobic	*Distichlis spicata*	Argentina: Buenos Aires	[47]
	Living, senescent, and decaying leaves	Saprobic	*Juncus roemerianus*	USA: Florida	[43]
**Beltraniaceae**					
*Beltrania querna* Harkn.	Decaying leaves	Saprobic	*Juncus roemerianus*	USA: Florida	[43]
**Hyponectriaceae**					
*Phragmitensis ellipsoidea* M.K.M. Wong, Goh and K.D. Hyde	Intertidal to aerial culms	Saprobic	*Phragmites australis*	China: Hong Kong	[115]
*Phragmitensis marina* M.K.M. Wong, Poon and K.D. Hyde	Decaying stems and leaf sheaths	Saprobic	*Phragmites australis*	China: Hong Kong	[41]
*Physalospora citogerminans* Kohlm., Volkm.-Kohlm. and O.E. Erikss.	Lower and upper parts of senescent culms	Saprobic	*Juncus roemerianus*	USA: North Carolina	[116]
**Sporocadaceae**					
*Discostroma* sp.	Living/decomposing leaf sheaths	Saprobic	*Phragmites australis*	Netherlands: Zeeland	[39]
*Pestalotiopsis juncestris* Kohlm. and Volkm.-Kohlm.	Senescent involucral leaves and culms	Saprobic	*Juncus roemerianus*	USA: North Carolina	[117]
*Pestalotiopsis planimi* (Vize) Steyaert	–	–	*Spartina alterniflora*	USA: Rhode Island	[61]
*Pestalotiopsis* sp.	Decaying stems and leaf sheaths	Saprobic	*Phragmites australis*	China: Hong Kong	[41]
**Coronophorales**					
**Ceratostomataceae**					
*Melanospora* sp.	Decaying leaves	Saprobic	*Juncus roemerianus*	USA: Florida	[43]
*Microthecium fimicola* (E.C. Hansen) Y. Marín, Stchigel, Guarro and Cano	–	–	*Elymus pungens*	UK	[38]
*Microthecium levitum* Udagawa and Cain	Dead leaves/culms	Saprobic	*Spartina alterniflora*	USA: Rhode Island	[61]
**Coronophorales genera *incertae sedis***					
*Papulaspora halima* Anastasiou	Living and decaying leaves	Saprobic	*Juncus roemerianus*	USA: Florida	[43]
*Papulosa amerospora* Kohlm. and Volkm.-Kohlm.	Senescent culms	Saprobic	*Juncus roemerianus*	USA: North Carolina	[118]
**Diaporthales**					
**Diaporthaceae**					
*Phomopsis* spp.	Senescent and decaying leaves	Saprobic	*Juncus roemerianus*	USA: Florida	[43]
	Decaying stems and leaf sheaths	Saprobic	*Phragmites australis*	China: Hong Kong	[41]
	–	–	*Spartina* sp.	–	[71]
**Gnomoniaceae**					
*Gnomonia salina* E.B.G. Jones (*probably a nomen dubiumand possibly a Halosarpheia species)*	–	Saprobic	*Spartina alterniflora*	USA: Connecticut	[36]
			*Spartina* spp.		[32,35]
	–	–	*Spartina townsendii*	UK	[35,65]
**Diaporthales *incertae sedis***					
*Botryodiplodia* sp.	Senescent and decaying leaves	Saprobic	*Juncus roemerianus*	USA: Florida	[43]
**Glomerellales**					
**Glomerellaceae**					
*Colletotrichum* sp.	Decaying stems and leaf sheaths	Saprobic	*Phragmites australis*	China: Hong Kong	[41]
**Plectosphaerellaceae**					
*Stachylidium bicolor* Link	Senescent leaves	Saprobic	*Juncus roemerianus*	USA: Florida	[43]
**Hypocreales**					
**Bionectriaceae**					
*Acremonium* spp.	Leaves	Saprobic	*Distichlis spicata*	Argentina: Buenos Aires	[47]
	Living, senescent, and decaying leaves	Saprobic	*Juncus roemerianus*	USA: Florida	[43]
	Decaying stems and leaf sheaths	Saprobic	*Phragmites australis*	China: Hong Kong	[41]
*Clonostachys rosea* (Link) Schroers, Samuels, Seifert and W. Gams	Leaves	Saprobic	*Spartina* sp.	Canada: Bay of Fundy	[48]
*Fusariella obstipa* (Pollack) S. Hughes	Decaying leaves	Saprobic	*Juncus roemerianus*	USA: Florida	[43]
*Gliomastix* spp.	Senescent and decaying leaves	Saprobic	*Juncus roemerianus*	USA: Florida	[43]
	Decaying stems and leaf sheaths	Saprobic	*Phragmites australis*	China: Hong Kong	[41]
*Hydropisphaera arenula* (Berk. and Broome) Rossman and Samuels	Living/decomposing leaf sheaths	Saprobic	*Phragmites australis*	Netherlands: Zeeland	[39]
*Hydropisphaera erubescens* (Roberge ex Desm.) Rossman and Samuels	Decaying leaf blades	Saprobic	*Spartina alterniflora*	USA: Georgia	[56]
	–	–	*Spartina* spp.	–	[32]
**Clavicipitaceae**					
*Atkinsonella hypoxylon* (Peck) Diehl	–	–	*Spartina cynosuroides*	–	[68]
*Claviceps purpurea* (Fr.) Tul.	–	Saprobic	*Phragmites australis*	UK: England (Southampton Hampshire, Sussex, Oxon)	[119,120]
	Replaced seeds in the inflorescence, ovaries of the flowers	Saprobic, parasitic	*Spartina alterniflora*	USA: Rhode Island; Argentina	[36,61,68,73,121,122]
	–	Pathogenic	*Spartina anglica*	UK	[123]
	–	Saprobic, parasitic	*Spartina cynosuroides*	USA: New York, Florida, Mississippi	[44,68,121,124]
			*Spartina patens*	USA: Maryland, Mississippi	[44,68,124,125]
	–	–	*Spartina townsendii*	UK: England	[120,126]
	–	–	*Spartina* sp.	Argentina	[122]
*Claviceps* sp.	–	–	*Spartina foliosa*	USA: California	[127]
*Metarhizium anisopliae* (Metschn.) Sorokīn	Leaves	Saprobic	*Distichlis spicata*	Argentina: Buenos Aires	[47]
**Hypocreaceae**					
*Cladobotryum* sp.	Decaying leaves	Saprobic	*Juncus roemerianus*	USA: Florida	[43]
*Gliocladium* sp.	Senescent leaves	Saprobic	*Juncus roemerianus*	USA: Florida	[43]
*Trichoderma citrinum* (Pers.) Jaklitsch, W. Gams and Voglmayr	Leaves	Saprobic	*Spartina* sp.	Canada: Bay of Fundy	[48]
*Trichoderma* sp.	Decaying stems and leaf sheaths	Saprobic	*Phragmites australis*	China: Hong Kong	[41]
*Trichoderma viride* Pers.	Living, senescent, and decaying leaves	Saprobic	*Juncus roemerianus*	USA: Florida	[43]
**Nectriaceae**					
*Calonectria* sp.	–	–	*Elymus pungens*	UK	[38]
*Fusarium fujikuroi* Nirenberg	–	Saprobic	*Suaeda australis*	South Australia	[62]
*Fusarium graminearum* Schwabe	Living/decomposing leaf sheaths, stems	Saprobic	*Phragmites australis*	Netherlands: Zeeland	[39,40]
*Fusarium heterosporum* Nees and T. Nees	–	–	*Spartina maritima*	–	[128]
*Fusarium incarnatum (Desm.) Sacc*.	Leaves	Saprobic	*Distichlis spicata*	Argentina: Buenos Aires	[47]
*Fusarium oxysporum* Schltdl.	Leaves	Saprobic	*Distichlis spicata*	Argentina: Buenos Aires	[47]
	Leaves and roots	Saprobic	*Spartina* sp.	Canada: Bay of Fundy	[48]
*Fusarium poae (Peck) Wollenw*.	Leaves	Saprobic	*Distichlis spicata*	Argentina: Buenos Aires	[47]
*Fusarium solani* (Mart.) Sacc.	Decaying stems and leaf sheaths	Saprobic	*Phragmites australis*	China: Hong Kong	[41]
*Fusarium* spp.	Leaves	Saprobic	*Distichlis spicata*	Argentina: Buenos Aires	[47]
	Living, senescent, and decaying leaves	Saprobic	*Juncus roemerianus*	USA: Florida	[43]
	Living/decomposing leaf sheaths, stems	Saprobic	*Phragmites australis*	China: Hong Kong; Netherlands: Zeeland	[39,40,41]
	Leaf sheaths and blades, stem	Saprobic	*Spartina maritima*	Portugal: Algarve	[59]
*Gibberella* sp.	–	Saprobic	*Spartina alterniflora*	Argentina: Buenos Aires	[36]
*Nectria* sp.	Senescent and decaying leaves	Saprobic	*Juncus roemerianus*	USA: Florida	[43]
*Tubercularia pulverulenta* Speg.	–	–	*Sarcocornia perennis*	–	[35]
	–	–	*Salicornia europaea*	–	[35]
	–	Saprobic	Unidentified saltmarsh plants	USA: Mississippi	[58]
	–	–	*Sarcocornia fruticosa*	–	[35]
*Tubercularia* sp.	Decaying leaf blades	Saprobic	*Spartina alterniflora*	USA: Georgia	[56]
*Volutella ciliata* (Alb. and Schwein.) Fr.	Leaves	Saprobic	*Distichlis spicata*	Argentina: Buenos Aires	[47]
**Sarocladiaceae**					
*Sarocladium implicatum* (J.C. Gilman and E.V. Abbott) A. Giraldo, Gené and Guarro	Leaves	Saprobic	*Distichlis spicata*	Argentina: Buenos Aires	[47]
*Sarocladium* sp.	Decaying stems and leaf sheaths	Saprobic	*Phragmites australis*	China: Hong Kong	[41]
**Stachybotryaceae**					
*Albifimbria verrucaria* (Alb. and Schwein.) L. Lombard and Crous	Leaves	Saprobic	*Distichlis spicata*	Argentina: Buenos Aires	[47]
*Paramyrothecium roridum* (Tode) L. Lombard and Crous	Living, senescent, and decaying leaves	Saprobic	*Juncus roemerianus*	USA: Florida	[43]
*Stachybotrys chartarum* (Ehrenb.) S. Hughes	Senescent and decaying leaves	Saprobic	*Juncus roemerianus*	USA: Florida	[43]
*Stachybotrys cylindrosporus* C.N. Jensen	Decaying leaves	Saprobic	*Juncus roemerianus*	USA: Florida	[43]
*Stachybotrys echinatus* (Rivolta) G. Sm.	Senescent and decaying leaves	Saprobic	*Juncus roemerianus*	USA: Florida	[43]
*Stachybotrys kampalensis* Hansf.	Senescent leaves	Saprobic	*Juncus roemerianus*	USA: Florida	[43]
*Stachybotrys nephrosporus* Hansf.	Senescent and decaying leaves	Saprobic	*Juncus roemerianus*	USA: Florida	[43]
*Stachybotrys* spp.	Senescent and decaying leaves	Saprobic	*Juncus roemerianus*	USA: Florida	[43]
	Decaying stems and leaf sheaths	Saprobic	*Phragmites australis*	China: Hong Kong	[41]
	Decaying leaf blades	Saprobic	*Spartina alterniflora*	USA: Georgia	[56]
*Striaticonidium cinctum* (Corda) L. Lombard and Crous	Living/decomposing leaf sheaths	Saprobic	*Phragmites australis*	Netherlands: Zeeland	[39]
*Xepicula jollymannii* (N.C. Preston) L. Lombard and Crous	Senescent and decaying leaves	Saprobic	*Juncus roemerianus*	USA: Florida	[43]
**Hypocreales genera *incertae sedis***					
*Cephalosporium* spp.	Dead leaves/culms	Saprobic	*Spartina alterniflora*	USA: Rhode Island	[61]
**Lulworthiales**					
**Lulworthiaceae**					
*Cumulospora marina* I. Schmidt	Dead culm	Saprobic	*Phragmites australis*	Iraq, Egypt, Germany, Thailand	[129]
	–	–	*Spartina* spp.	–	[32]
*Halazoon fuscus* (I. Schmidt) Abdel-Wahab, K.L. Pang, Nagah., Abdel-Aziz and E.B.G. Jones	Decaying rhizomes	Saprobic	*Phragmites australis*	France, Germany, Japan	[35,130]
	Rhizomes and culms	Saprobic	*Phragmites* sp.	Sweden	[87]
*Halazoon melhae Abdel-Aziz*, *Abdel-Wahab and Nagahama*	Decaying stem	Saprobic	*Phragmites australis*	Egypt: Port Said	[130]
*Lulworthia floridana* Meyers	–	Saprobic	*Spartina alterniflora*	USA: North Carolina, Rhode Island	[20,131]
*Lulworthia medusa* (Ellis and Everh.) Cribb and J.W. Cribb	–	–	*Elymus pungens*	UK	[38]
	–	Saprobic	*Spartina cynosuroides*	USA: New Jersey	[89,132]
	–		*Spartina* spp.	USA: New Jersey	[32,89]
	Stems	Saprobic	*Spartina townsendii*	UK: England (Wales); USA: Virginia, North Carolina, South Carolina, Florida, Texas	[38,49,71,72,89,132,133,134]
*Lulworthia* spp.	–	–	*Elymus pungens*	–	[35]
	–	–	*Juncus roemerianus*	–	[35,36]
	Dead culms	Saprobic	*Spartina alterniflora*	Argentina: Buenos Aires; USA: Rhode Island, North Carolina	[35,36,61,73,74]
	–	–	*Spartina cynosuroides*	–	[35]
	–	Saprobic	*Spartina* sp.	Argentina: Buenos Aires; Canada; USA: Maine, North Carolina	[36]
	–	–	*Spartina townsendii*	–	[35]
	Leaf sheaths and blades, stem	Saprobic	*Spartina maritima*	Portugal: Alentejo, Lisbon, Algarve, Centro	[31,54,59,63]
*Moleospora maritima* Abdel-Wahab, Abdel-Aziz and Nagah.	Decayed stems	Saprobic	*Phragmites australis*	Egypt: Port Said	[130]
**Magnaporthales**					
**Ceratosphaeriaceae**					
*Ceratosphaeria* sp.	Senescent leaves	Saprobic	*Juncus roemerianus*	USA: Florida	[43]
**Magnaporthaceae**					
*Buergenerula spartinae* Kohlm. and R.V. Gessner	Lower stem and leaf sheath during the growth phase of the plant/living and dead; decaying leaf blades	Saprobic, parasitic	*Spartina alterniflora*	USA: Alabama, Rhode Island, Maine, New Hampshire, Connecticut, Mississippi, New Jersey, Virginia, North Carolina, Florida, Georgia	[20,35,36,55,56,58,61,73,74,82,92]
	Leaves	Saprobic	*Spartina* spp.	Canada: Bay of Fundy; USA: South Carolina; UK	[32,35,36,48,65] this study
	Leaf sheaths and blades, stem	Saprobic	*Spartina maritima*	Portugal: Alentejo, Lisbon, Algarve, Centro	[31,54,59]
*Gaeumannomyces* sp.	Decaying stems and leaf sheaths	Saprobic	*Phragmites australis*	China: Hong Kong	[41]
*Kohlmeyeriopsis medullaris* (Kohlm., Volkm.-Kohlm. and O.E. Erikss.) Klaubauf, M.-H. Lebrun and Crous	Lower parts of senescent culms	Saprobic	*Juncus roemerianus*	USA: North Carolina	[97,135]
*Utrechtiana roumeguerei* (Cavara) Videira and Crous	Living/decomposing leaf blades and sheaths	Saprobic	*Phragmites australis*	Netherlands: Zeeland	[39,50]
**Pseudohalonectriaceae**					
*Pseudohalonectria falcata* Shearer	Decaying stems and leaf sheaths	Saprobic	*Phragmites australis*	China: Hong Kong	[41]
*Pseudohalonectria halophila* Kohlm. and Volkm.-Kohlm.	Fragments of leaves and culms in the wrack	Saprobic	*Juncus roemerianus*	USA: North Carolina	[105]
**Meliolales**					
**Meliolaceae**					
*Meliola arundinis* Pat.	–	–	*Phragmites australis*	Australia: Queensland	[62]
**Microascales**					
**Halosphaeriaceae**					
*Aniptodera chesapeakensis* Shearer and M.A. Mill.	Dead leaves	Saprobic	*Juncus roemerianus*	USA: North Carolina	[35]
	Decaying stems and leaf sheaths	Saprobic	*Phragmites australis*	China: Hong Kong	[41]
	–	–	*Spartina alterniflora*	–	[35]
	–	–	*Spartina* spp.	–	[32]
	Leaf sheaths and blades, stem	Saprobic	*Spartina maritima*	Portugal: Alentejo, Algarve, Centro	[59,63]
*Aniptodera juncicola Volkm*.-Kohlm. and Kohlm.	Dead standing culms of	Saprobic	*Juncus roemerianus*	India: Kerala, West Bengal, Tamil Nadu; USA: North Carolina	[52,136]
*Aniptodera phragmiticola* O. K. Poon et K. D. Hyde	Decaying stems and leaf sheaths	Saprobic	*Phragmites australis*	China: Hong Kong	[41]
*Ceriosporopsis halima* Linde	–	–	*Arundo donax*	–	[35]
	Submerged seeds	Saprobic	*Spartina alterniflora*	USA	[137]
	–	–	*Spartina* spp.	–	[32]
	–	–	*Spartina townsendii*	UK	[35,38]
	Stem	Saprobic	*Spartina maritima*	Portugal: Alentejo	[63]
*Cirrenalia macrocephala* (Kohlm.) Meyers and R.T. Moore	–	–	*Ammophila arenaria*	–	[35]
	Decaying culms	Saprobic	*Juncus roemerianus*	USA: Florida	[43]
	Decomposing culms, submerged seeds	Saprobic	*Spartina alterniflora*	USA: Rhode Island	[35,61,137]
	–	–	*Spartina* spp.	–	[32]
	Stem	Saprobic	*Spartina maritima*	Portugal: Alentejo	[63]
*Cirrenalia pseudomacrocephala* Kohlm.	Senescent leaves	Saprobic	*Juncus roemerianus*	USA: Florida	[43]
*Corollospora maritima* Werderm.	Submerged seeds, decomposing culms	Saprobic	*Spartina alterniflora*	USA: Rhode Island	[20,35,61,137]
	–	–	*Spartina* spp.	–	[32]
	Stem	Saprobic	*Spartina maritima*	Portugal: Alentejo	[63]
	–	Saprobic	Unidentified saltmarsh plants	USA: Mississippi	[58]
*Corollospora ramulosa* (Meyers and Kohlm.) E.B.G. Jones and Abdel-Wahab	–	Saprobic	Unidentified saltmarsh plants	USA: Mississippi	[58]
	–	Saprobic	*Zostera marina*	USA: North Carolina	[74]
*Haligena elaterophora* Kohlm.	–	–	*Spartina alterniflora*	–	[35]
	–	–	*Spartina tonwsendii*	UK	[38]
	–	–	*Spartina* spp.	–	[32]
*Halosarpheia culmiperda* Kohlm., Volkm.-Kohlm. and O.E. Erikss.	Lower parts of senescent culms	Saprobic	*Juncus roemerianus*	USA: North Carolina	[97]
*Halosarpheia* sp.	Stem	Saprobic	*Spartina maritima*	Portugal: Alentejo	[63]
*Halosarpheia viscosa* I. Schmidt ex Shearer and J.L. Crane	Decaying leaf blades	Saprobic	*Spartina alterniflora*	USA: Georgia	[56]
	–	Saprobic	*Spartina maritima*	Portugal: Lisbon	[54]
*Halosphaeria appendiculata* Linder	–	–	*Arundo donax*	–	[35]
*Halosphaeria* sp.	Submerged seeds	Saprobic	*Spartina alterniflora*	USA	[137]
*Lautisporopsis circumvestita* (Kohlm.) E.B.G. Jones, Yusoff and S.T. Moss	–	–	*Arundo donax*	–	[35]
*Lignincola laevis* Höhnk	–	–	*Elymus pungens*	–	[35]
	–	Saprobic	*Spartina* spp.	USA: North Carolina	[32,138]
	–	–	*Spartina townsendii*	–	[35]
	Stem	Saprobic	*Spartina maritima*	Portugal: Alentejo	[63]
*Magnisphaera spartinae* (E.B.G. Jones) J. Campb., J.L. Anderson and Shearer	–	–	*Elymus farctus*	–	[35]
	–	–	*Elymus pungens*	–	[35]
	Living/decomposing stems	Saprobic	*Phragmites australis*	Netherlands: Zeeland	[40]
	–	Saprobic	*Spartina alterniflora*	USA: Rhode Island	[20,35,61]
	–		*Spartina* spp.		[32]
	–	Saprobic	*Spartina patens*	USA: Rhode Island	[36]
	Stem	Saprobic	*Spartina townsendii*	UK: Wales	[35,139]
	–		*Typha* sp.		[35]
*Nais inornata* Kohlm.	Decomposing culms	Saprobic	*Spartina alterniflora*	USA: Rhode Island	[20,35,61]
			*Spartina* spp.		[32]
*Natantispora unipolaris* K.L. Pang, S.Y. Guo and E.B.G. Jones	Dead stem	Saprobic	*Phragmites australis*	Taiwan: Nankunshen	[140]
*Natantispora retorquens* (Shearer and J.L. Crane) J. Campb., J.L. Anderson and Shearer	Leaf sheaths and blades, stem	Saprobic	*Spartina maritima*	Portugal: Alentejo, Lisbon, Algarve, Centro	[31,54,59,63]
*Oceanitis unicaudata* (E.B.G. Jones and Camp.-Als.) J. Dupont and E.B.G. Jones	Decaying stems and leaf sheaths	Saprobic	*Phragmites australis*	China: Hong Kong	[41]
	Stem	Saprobic	*Spartina maritima*	Portugal: Alentejo	[63]
*Panorbis viscosus* (I. Schmidt) J. Campb., J.L. Anderson and Shearer	Leaf sheaths and blades, stem	Saprobic	*Spartina maritima*	Portugal: Alentejo, Algarve	[59,63]
*Remispora hamata* (Höhnk) Kohlm.	–	–	*Elymus pungens*	UK	[35,38]
	Senescent and decaying leaves	Saprobic	*Juncus roemerianus*	USA: Florida	[43]
	Living/decomposing leaf blades and sheaths, stems	Saprobic	*Phragmites australis*	Netherlands: Zeeland	[39,40,50]
	–	Saprobic	*Phragmites* sp.	Sweden	[87]
	Dead leaves	Saprobic	*Spartina alterniflora*	USA: Rhode Island, Maine, Florida	[20,35,36,61,73]
	–	Saprobic	*Spartina patens*	USA: Rhode Island	[36]
	–	Saprobic	*Spartina* sp.	USA: North Carolina; Argentina: Buenos Aires	[36,138]
	–	–	*Spartina townsendii*	–	[35]
	–	–	*Typha* sp.	–	[35]
*Remispora trullifera* Kohlm.	Leaf sheaths and blades, stem	Saprobic	*Spartina maritima*	Portugal: Centro	[59]
*Tirispora unicaudata* E.B.G. Jones and Vrijmoed	Stem	Saprobic	*Spartina maritima*	Portugal: Alentejo	[63]
**Microascaceae**					
*Scopulariopsis* spp.	Living, senescent, and decaying leaves	Saprobic	*Juncus roemerianus*	USA: Florida	[43]
**Myrmecridiales**					
**Myrmecridiaceae**					
*Myrmecridium schulzeri* (Sacc.) Arzanlou, W. Gams and Crous	Leaves	Saprobic	*Distichlis spicata*	Argentina: Buenos Aires	[47]
**Ophiostomatales**					
**Ophiostomataceae**					
*Sporothrix* sp.	Senescent leaves	Saprobic	*Juncus roemerianus*	USA: Florida	[43]
**Phomatosporales**					
**Phomatosporaceae**					
*Phomatospora bellaminuta* Kohlm., Volkm.-Kohlm. and O.E. Erikss.	Lower parts of senescent culms	Saprobic	*Juncus roemerianus*	USA: North Carolina	[116]
*Phomatospora berkeleyi* Sacc.	Living/decomposing leaf blades and sheaths, stems	Saprobic	*Phragmites australis*	Netherlands: Zeeland	[39,40,50]
*Phomatospora dinemasporium* J. Webster	Decaying stems and leaf sheaths, stems	Saprobic	*Phragmites australis*	China: Hong Kong; Netherlands: Zeeland	[40,41]
	Dead leaves	Saprobic	*Phragmites* sp.	South Australia	[62]
	–	–	*Spartina townsendii*	UK	[38]
*Phomatospora phragmiticola* Poon and K.D. Hyde	Decaying stems and leaf sheaths	Saprobic	*Phragmites australis*	China: Hong Kong	[41]
*Phomatospora* spp.	Senescent and decaying leaves	Saprobic	*Juncus roemerianus*	USA: Florida	[43]
	Living/decomposing leaf sheaths, stems	Saprobic	*Phragmites australis*	Netherlands: Zeeland	[39,40]
**Phyllachorales**					
**Phyllachoraceae**					
*Phyllachora graminis* (Pers.) Fuckel	–	–	*Elymus pungens*	UK	[38]
	–	Saprobic, pathogenic	*Spartina alterniflora*	USA: Massachusetts	[44]
	–	–	*Spartina cynosuroides*	–	[68]
*Phyllachora cynodontis* Niessl.	–	Saprobic, pathogenic	*Spartina alterniflora*	USA	[68]
	–	Saprobic, pathogenic	*Spartina foliosa*	USA: California	[44,112,141]
*Phyllachora paludicola* Kohlm. and Volkm.-Kohlm.	Dead leaves (lower half of standing culms)	Saprobic	*Spartina alterniflora*	USA: Florida, Georgia, North Carolina, Maryland, Delaware	[142]
*Phyllachora sylvatica* Sacc. and Speg.	–	Saprobic	*Spartina patens*	USA: South Carolina	[141]
**Savoryellales**					
**Savoryellaceae**					
*Savoryella paucispora* (Cribb and J.W. Cribb) J. Koch	–	–	*Elymus pungens*	–	[35]
	–	–	*Juncus roemerianus*	–	[35]
	–	–	*Spartina alterniflora*	–	[35]
	–	–	*Spartina* sp.	–	[35]
	–	–	*Spartina townsendii*	–	[35]
**Sordariales**					
**Chaetomiaceae**					
*Achaetomium* sp.	Decaying leaves	Saprobic	*Juncus roemerianus*	USA: Florida	[43]
*Chaetomium elatum* Kunze	–	–	*Elymus pungens*	UK	[38]
	–	–	*Puccinellia maritima*	UK	[38]
	–	–	*Spartina townsendii*	UK	[38]
	–	–			
*Chaetomium globosum* Kunze	–	–	*Elymus pungens*	UK	[38]
	Decaying stems and leaf sheaths	Saprobic	*Phragmites australis*	China: Hong Kong	[41]
	–	–	*Puccinellia maritima*	UK	[38]
	–	–	*Spartina townsendii*	UK	[38]
*Chaetomium spirale* Zopf	–	–	*Elymus pungens*	UK	[38]
*Chaetomium thermophilum* La Touche	–	–	*Elymus pungens*	UK	[38]
	–	–	*Puccinellia maritima*	UK	[38]
	–	–	*Spartina townsendii*	UK	[38]
*Chaetomium* sp.	Stem	Saprobic	*Typha* sp.	UK	This study
*Corynascus sepedonium* (C.W. Emmons) Arx	–	–	*Elymus pungens*	UK	[38]
	–	–	*Puccinellia maritima*	UK	[38]
	–	–	*Spartina townsendii*	UK	[38]
*Dichotomopilus funicola* (Cooke) X.Wei Wang and Samson	–	–	*Elymus pungens*	UK	[38]
	–	–	*Spartina alterniflora*	USA: Rhode Island	[61]
	–	–	*Spartina townsendii*	UK	[38]
*Dichotomopilus indicus* (Corda) X.Wei Wang and Samson	–	–	*Elymus pungens*	UK	[38]
*Humicola* sp.	Living, senescent, and decaying leaves	Saprobic	*Juncus roemerianus*	USA: Florida	[43]
	Decaying stems and leaf sheaths	Saprobic	*Phragmites australis*	China: Hong Kong	[41]
*Thermothielavioides terrestris* (Apinis) X. Wei Wang and Houbraken	–	–	*Elymus pungens*	UK	[38]
	–	–	*Puccinellia maritima*	UK	[38]
*Trichocladium constrictum* I. Schmidt	Stem	Saprobic	*Spartina maritima*	Portugal: Alentejo	[63]
*Trichocladium crispatum* (Fuckel) X. Wei Wang and Houbraken	–	–	*Elymus pungens*	UK	[38]
	–	–	*Spartina townsendii*	UK	[38]
**Lasiosphaeriaceae**	**–**	**–**			
*Schizothecium hispidulum* (Speg.) N. Lundq.	Living/decomposing leaf sheaths	Saprobic	*Phragmites australis*	Netherlands: Zeeland	[39]
*Zopfiella latipes* (N. Lundq.) Malloch and Cain	Decaying stems and leaf sheaths	Saprobic	*Phragmites australis*	China: Hong Kong	[41]
**Sordariaceae**					
*Neurospora calospora* (Mouton) Dania García, Stchigel and Guarro	–	–	*Elymus pungens*	UK	[38]
*Sordaria fimicola* (Roberge ex Desm.) Ces. and De Not.	–	–	*Elymus pungens*	UK	[38]
	Leaves	Saprobic	*Distichlis spicata*	Argentina: Buenos Aires	[47]
	–	–	*Puccinellia maritima*	UK	[38]
	–	–	*Spartina townsendii*	UK	[38]
**Sordariomycetes families *incertae sedis***					
*Koorchaloma galateae* Kohlm. and Volkm.-Kohlm.	Senescent culms	Saprobic	*Juncus roemerianus*	USA: North Carolina	[117]
*Koorchaloma spartinicola* V.V. Sarma, S.Y. Newell and K.D. Hyde	Decaying leaf blades	Saprobic	*Spartina alterniflora*	USA: Georgia	[56]
*Koorchaloma* sp.	Decaying leaf blades	Saprobic	*Spartina alterniflora*	USA: Georgia	[56]
*Lautospora simillima* Kohlm., Volkm.-Kohlm. and O.E. Erikss.	Lower parts of senescent, soft culms	Saprobic	*Juncus roemerianus*	USA: North Carolina	[78]
**Sordariomycetes genera *incertae sedis***					
*Aquamarina speciosa* Kohlm., Volkm.-Kohlm. and O.E. Erikss.	Senescent culms		*Juncus roemerianus*	USA: Georgia, North Carolina, Virginia	[77]
*Aropsiclus junci* (Kohlm. and Volkm.-Kohlm.) Kohlm. and Volkm.-Kohlm.	Senescent culms	Saprobic	*Juncus roemerianus*	USA: North Carolina	[143]
*Zalerion maritima* (Linder) Anastasiou	Basal area of the sheath	Saprobic	*Spartina densiflora*	Argentina: Buenos Aires	[64]
	–	–	*Spartina* spp.	–	[32]
*Ellisembia* sp.	Decaying stems and leaf sheaths	Saprobic	*Phragmites australis*	China: Hong Kong	[41]
**Torpedosporales**					
**Juncigenaceae**					
*Juncigena adarca* Kohlm., Volkm.-Kohlm. and O.E. Erikss.	Senescent leaves	Saprobic	*Juncus roemerianus*	USA: North Carolina	[76]
*Moheitospora adarca* (Kohlm., Volkm.-Kohlm. and O.E. Erikss.) Abdel-Wahab, Abdel-Aziz and Nagah	Stems	Saprobic	*Juncus roemerianus*	USA	[130]
*Moheitospora fruticosae* Abdel-Wahab, Abdel-Aziz and Nagah.	Decayed stems	Saprobic	*Suaeda vermiculata*	Egypt: Alexandria	[130]
*Torpedospora radiata* Meyers	–	Saprobic	Unidentified saltmarsh plants	USA: Mississippi	[58]
**Tracyllalales**					
**Tracyllaceae**					
*Tracylla spartinae* (Peck) Tassi	–	Saprobic, pathogenic	*Spartina patens*	USA: Mississippi	[44,68]
**Xylariales**					
**Diatrypaceae**					
*Cryptovalsa suaedicola* Spooner	Dead twigs	Saprobic	*Suaeda vermiculata*	UK: Great Britain	[144]
*Halocryptovalsa salicorniae* Dayar. and K.D. Hyde	Dead stem	Saprobic	*Salicornia* sp.	Thailand: Prachuap Khiri Khan	[145]
**Xylariaceae**					
*Anthostomella atroalba* Kohlm., Volkm.-Kohlm. and O.E. Erikss.	Senescent leaves	Saprobic	*Juncus roemerianus*	USA: North Carolina	[60]
*Anthostomella lugubris* (Roberge ex Desm.) Sacc.	–	–	*Elymus pungens*	UK	[38]
*Anthostomella phaeosticta* (Berk.) Sacc.	–	–	*Elymus pungens*	UK	[38]
*Anthostomella poecila* Kohlm., Volkm.-Kohlm. and O.E. Erikss.	Lower and upper parts of senescent culms, decaying leaves	Saprobic	*Juncus roemerianus*	USA: Alabama, Mississippi, North Carolina	[55,58,116]
*Anthostomella punctulata* (Roberge ex Desm.) Sacc.	Living/decomposing leaf blades and sheaths	Saprobic	*Phragmites australis*	Netherlands: Zeeland	[39,50]
*Anthostomella semitecta* Kohlm., Volkm.-Kohlm. and O.E. Erikss.	Senescent culms	–	*Juncus roemerianus*	USA: North Carolina	[116]
*Anthostomella spissitecta* Kohlm. and Volkm.-Kohlm.	Leaf sheaths of senescent culms	Saprobic	*Spartina alterniflora*, *S. densiflora*.	USA: Connecticut, Florida, North Carolina, Rhode Island; Argentina: Buenos Aires	[32]
	–	–	*Spartina* sp.	–	[32]
	Leaf sheaths and blades, stem	Saprobic	*Spartina maritima*	Portugal: Algarve	[59]
*Anthostomella* spp.	–	–	*Elymus pungens*	UK	[38]
	–	Saprobic	*Spartina alterniflora*	USA: Connecticut, Florida, North Carolina, Rhode Island; Argentina	[36,61]
	–	–	*Spartina townsendii*	UK	[38]
*Anthostomella torosa* Kohlm. and Volkm.-Kohlm.	Senescent culms (restricted to short culms)	Saprobic	*Juncus roemerianus*	USA: North Carolina	[32]
*Geniculosporium* sp.	Living, senescent, and decaying leaves	Saprobic	*Juncus roemerianus*	USA: Florida	[43]
*Rosellinia* sp.	Dead leaves/culms	Saprobic	*Spartina alterniflora*	USA: Rhode Island	[61]
*Virgaria nigra* (Link) Nees	Senescent leaves	Saprobic	*Juncus roemerianus*	USA: Florida	[43]
**Zygosporiaceae**					
*Zygosporium gibbum* (Sacc., M. Rousseau and E. Bommer) S. Hughes	Decaying leaves	Saprobic	*Juncus roemerianus*	USA: Florida	[43]
*Zygosporium masonii* S. Hughes	Decaying leaves	Saprobic	*Juncus roemerianus*	USA: Florida	[43]
*Zygosporium* sp.	Decaying leaves	Saprobic	*Juncus roemerianus*	USA: Florida	[43]
**Xylariales genera *incertae sedis***					
*Circinotrichum maculiforme* Nees	Decaying leaves	Saprobic	*Juncus roemerianus*	USA: Florida	[43]
**Xylariomycetidae family *incertae sedis***					
**Cainiaceae**					
*Atrotorquata lineata* Kohlm. and Volkm.-Kohlm.	Senescent culms	Saprobic	*Juncus roemerianus*	USA: North Carolina	[104]
		Saprobic	Unidentified saltmarsh plant	USA: Mississippi	[58]
**Ascomycota genera *incertae sedis***					
*Asteromyces cruciatus* C. Moreau and Moreau ex Hennebert	–	–	*Agropyron* sp.	–	[35]
	–	–	*Ammophila arenaria*	–	[35]
	–	–	*Spartina* spp.	–	[32,35]
	–	Saprobic	*Zostera* sp.	USA: California	[74]
*Cremasteria cymatilis* Meyers and R.T. Moore Nomen dubium	Senescent leaves	Saprobic	*Juncus roemerianus*	USA: Florida	[43]
*Cytoplacosphaeria phragmiticola* Poon and K.D. Hyde	Decaying stems and leaf sheaths	Saprobic	*Phragmites australis*	China: Hong Kong	[41]
*Cytoplacosphaeria rimosa* (Oudem.) Petr.	Living/decomposing leaf sheaths, stems	Saprobic	*Phragmites australis*	Netherlands: Zeeland	[39,40]
*Cytosporina* sp.	Living, senescent, and decaying leaves	Saprobic	*Juncus roemerianus*	USA: Florida	[43]
*Didymosamarospora euryhalina* T.W. Johnson and H.S. Gold	Culms	Saprobic	*Juncus roemerianus*	USA: North Carolina	[146]
*Haplobasidion lelebae* Sawada ex M.B. Ellis	Living, senescent, and decaying leaves	Saprobic	*Juncus roemerianus*	USA: Florida	[43]
*Hymenopsis chlorothrix* Kohlm. and Volkm.-Kohlm.	Senescent culms	Saprobic	*Juncus roemerianus*	USA: North Carolina	[147]
*Hyphopolynema juncatile* Kohlm. and Volkm.-Kohlm.	Senescent leaves	Saprobic	*Juncus roemerianus*	USA: North Carolina	[148]
*Kolletes undulatus* Kohlm. and Volkm.-Kohlm.	Senescent leaves and culms	Saprobic	*Juncus roemerianus*	USA: North Carolina	[105]
*Minimidochium parvum* Cabello, Aramb. and Cazau	Leaves	Saprobic	*Distichlis spicata*	Argentina: Buenos Aires	[47]
*Monodictys pelagica* (T. Johnson) E.B.G. Jones	–	–	*Juncus* sp.	–	[35]
	Decomposing culms	Saprobic	*Spartina alterniflora*	USA: Rhode Island	[20,35,61,73]
	–	–	*Spartina* spp.	–	[32]
*Neottiospora* sp.	Living, senescent, and decaying leaves	Saprobic	*Juncus roemerianus*	USA: Florida	[43]
*Octopodotus stupendus* Kohlm. and Volkm.-Kohlm.	Dead leaves (lower half of standing culms)	Saprobic	*Spartina alterniflora*	USA: North Carolina	[142]
*Pycnodallia dupla* Kohlm. and Volkm.-Kohlm.	Senescent inflorescences (involucral leaves and branchlets)	Saprobic	*Juncus roemerianus*	USA: North Carolina	[147]
*Sphaeronaema* sp.	Senescent leaves	Saprobic	*Juncus roemerianus*	USA: Florida	[43]
*Stauronema* sp.	Decaying stems and leaf sheaths	Saprobic	*Phragmites australis*	China: Hong Kong	[41]
*Tetranacriella papillata* Kohlm. and Volkm.-Kohlm.	Senescent leaves	Saprobic	*Juncus roemerianus*	USA: North Carolina	[117]
*Tetranacrium* sp.	Decaying stems and leaf sheaths	Saprobic	*Phragmites australis*	China: Hong Kong	[41]
*Zythia* spp.	Living, senescent, and decaying leaves	Saprobic	*Juncus roemerianus*	USA: Florida	[43]
*Psammina* sp.	Senescent leaves	Saprobic	*Juncus roemerianus*	USA: Florida	[43]
					
**BASIDIOMYCOTA**					
**AGARICOMYCETES**					
**Agaricales**					
**Niaceae**					
*Merismodes bresadolae* (Grelet) Singer	Living/decomposing stems	Saprobic	*Phragmites australis*	Netherlands: Zeeland	[40]
*Nia globispora* Barata and Basilio	Stem	Saprobic	*Spartina maritima*	Portugal: Alentejo	[63]
*Nia vibrissa* R.T. Moore and Meyers	Old stem	Saprobic	*Spartina alterniflora*	USA: North Carolina	[35,149]
	–	Saprobic	*Spartina* spp.	USA: North Carolina	[32,150]
	Stem	Saprobic	*Spartina maritima*	Portugal: Alentejo	[63]
**AGARICOSTILBOMYCETES**					
**Agaricostilbales**					
**Chionosphaeraceae**					
*Stilbum* sp.	Decaying leaves	Saprobic	*Juncus roemerianus*	USA: Florida	[43]
					
**BARTHELETIOMYCETES**					
**Sebacinales**					
**Sebacinaceae**					
*Chaetospermum camelliae* Agnihothr.	Decaying stems and leaf sheaths	Saprobic	*Phragmites australis*	China: Hong Kong	[41]

**MICROBOTRYOMYCETES**					
**Sporidiobolales**					
**Sporidiobolaceae**					
*Sporobolomyces roseus* Kluyver and C.B. Niel	Leaves	Saprobic	*Spartina* sp.	Canada: Bay of Fundy	[48]
*Sporobolomyces* spp.	Living/decomposing leaf blades and sheaths	Saprobic	*Phragmites australis*	Netherlands: Zeeland	[39,50]
				
**PUCCINIOMYCETES**					
**Pucciniales**					
**Pucciniaceae**					
*Puccinia distichlidis* Ellis and Everh.	–	–	*Distichlis spicata*	USA	[151]
*Puccinia magnusiana* Körn.	Living/decomposing leaf blades and sheaths	Saprobic	*Phragmites australis*	Netherlands: Zeeland	[39,50]
*Puccinia phragmitis* (Schumach.) Tul.	Living/decomposing leaf blades and sheaths	Saprobic	*Phragmites australis*	Netherlands: Zeeland	[39,50]
*Puccinia sparganioidis* Ellis and Barthol.	–	Saprobic, parasitic	*Spartina alterniflora*	USA: Maine, New Hampshire, Massachusetts, Rhode Island, Delaware, Virginia, North Carolina, Florida, Mississippi	[36,44,68,73,152]
	–	–	*Spartina cynosuroides*	USA: New Jersey, Delaware, Maryland, South Carolina, Florida, Louisiana	[44,68,153]
	–	Saprobic, pathogenic	*Spartina patens*	USA: Connecticut, Maryland, New Jersey, New York	[44,68,153]
*Uromyces acuminatus* Arthur	–	Saprobic, pathogenic	*Spartina alterniflora*	USA: Maine, New Hampshire, Massachusetts, Connecticut, New York, New Jersey, Delaware, Maryland, Florida	[44,68,152]
	–	Saprobic, pathogenic	*Spartina cynosuroides*	USA: Florida	[44,68,153]
	–	Saprobic	*Spartina patens*	USA: Connecticut, Delaware, Florida, Maine, Maryland, Massachusetts, New Hampshire, New Jersey,	[44,68]
*Uromyces argutus* F. Kern	–	Saprobic, pathogenic	*Spartina alterniflora*	France; USA: Florida	[44,68,152]
*Uromyces salicorniae* (DC.) de Bary	–	–	*Salicornia* sp.	South Australia	[95]
**Pucciniales genera *incertae sedis***					
*Aecidium suaedae* Thüm.	Leaves	–	*Suaeda verae*	Egypt	[154]
					
**TREMELLOMYCETES**					
**Tremellales**					
**Tremellaceae**					
*Tremella spicifera* Van Ryck., Van de Put and P. Roberts	Living/decomposing leaf sheaths and stems	Saprobic	*Phragmites australis*	Netherlands: Zeeland	[39,40]
					
**USTILAGINOMYCETES**					
**Ustilaginales**					
**Ustilaginaceae**					
*Tranzscheliella distichlidis* (McAlpine) Vánky	–	Pathogenic	*Distichlis spicata*	Australia: Victoria	[155]
**Ustilaginales genera *incertae sedis***					
*Parvulago marina* (Durieu) R. Bauer, M. Lutz, Piątek, Vánky and Oberw.	–	–	*Eleocharis parvula*	Finland, France, Germany, UK, Norway, Sweden	[156]
**Urocystidales**					
**Urocystidaceae**					
*Flamingomyces ruppiae* (Feldmann) R. Bauer, M. Lutz, Piątek, Vánky and Oberw.	–	Parasitic	*Ruppia maritima*	France	[156]
					
**MUCOROMYCOTA**					
**MUCOROMYCETES**					
**Mucorales**					
**Choanephoraceae**					
*Blakeslea trispora* Thaxt.	Senescent and decaying leaves	Saprobic	*Juncus roemerianus*	USA: Florida	[43]
**Mucoraceae**					
*Mucor* sp.	Senescent leaves	Saprobic	*Juncus roemerianus*	USA: Florida	[43]
	Roots	Saprobic	*Spartina* sp.	Canada: Bay of Fundy	[48]
**Rhizopodaceae**					
*Rhizopus stolonifer* (Ehrenb.) Vuill.	Stems	Saprobic	*Spartina townsendii*	UK: England	[49]
**Syncephalastraceae**					
*Syncephalastrum racemosum* Cohn ex J. Schröt.	Living and senescent leaves	Saprobic	*Juncus roemerianus*	USA: Florida	[43]

## Data Availability

Not applicable.
